# Enhancing the Pharmacological Properties of Triterpenes Through Acetylation: An Anticancer and Antioxidant Perspective

**DOI:** 10.3390/molecules30122661

**Published:** 2025-06-19

**Authors:** Barbara Bednarczyk-Cwynar, Piotr Ruszkowski, Andrzej Günther, Szymon Sip, Katarzyna Bednarek-Rajewska, Przemysław Zalewski

**Affiliations:** 1 Department of Organic Chemistry, Faculty of Pharmacy, Poznan University of Medical Sciences, Collegium Pharmaceuticum 2, (CP.2), Rokietnicka Str. 3, 60-806 Poznan, Poland; andrzej.gunther@me.pl; 2 Center of Innovative Pharmaceutical Technology (CITF), Rokietnicka Str. 3, 60-806 Poznan, Poland; 3 Department of Pharmacology, Faculty of Pharmacy, Poznan University of Medical Sciences, Collegium Pharmaceuticum 1 (CP.1), Rokietnicka Str. 3, 60-806 Poznan, Poland; pruszkowski@gmail.com; 4 Department of Pharmacognosy and Biomaterials, Faculty of Pharmacy, Poznan University of Medical Sciences, Collegium Pharmaceuticum 1 (CP.1), Rokietnicka Str. 3, 60-806 Poznan, Poland; szymonsip@ump.edu.pl (S.S.); pzalewski@ump.edu.pl (P.Z.); 5 Department of Clinical Pathology, Faculty of Medical Sciences, Poznan University of Medical Sciences, Przybyszewskiego Str. 49, 60-355 Poznan, Poland; krajewska@ump.edu.pl

**Keywords:** oleanolic acid, erythrodiol, allobetulin, betulinic acid, betulin, lupeol, acyl derivatives of triterpenes, cytotoxic activity, Selectivity Index, molecular docking, antioxidant activity, SAR, ADMETox

## Abstract

This paper presents the influence of acetylation on the cytotoxic and antioxidant activity of natural triterpenes. Oleanolic acid, betulin, betulinic acid and other triterpenes have been modified to improve their pharmacological properties. Acylation of the hydroxyl group at the C-3 position showed significant changes in biological activity, in particular against cancer cell lines such as HeLa, A-549, MCF-7, PC-3 and SKOV-3, with the highest IC_50_ results for acetyloleanolic acid (**1b**) and acetylbetulinic acid (**4b**). Docking results showed that all compounds tested demonstrated the ability to bind to pockets (C1–C5) of the p53 Y220 protein, obtaining different Vina score values. The strongest binding was observed for compound **2b** in the C3 pocket (−10.1 kcal × mol^−1^), while in the largest C1 pocket, the best result was achieved by compound **5b** (−9.1 kcal × mol^−1^). Moreover, antioxidant studies using the CUPRAC and DPPH tests showed significant differences in the mechanisms of action of the compounds depending on the structure. The analyses of ADMETox confirmed the favorable pharmacokinetic profile and low toxicity of most of the tested derivatives. The results suggest that acetylated triterpenes, especially **1b** and **4b**, have great potential as anticancer drug candidates, requiring further research on their cytotoxic activity and structural modifications.

## 1. Introduction

Thanks to their unique biological and pharmacological properties, triterpenes have great research potential in the therapy and prevention of cancer and other diseases. Being a large group of natural compounds, they have a broad spectrum of chemical structures, which allow for diverse biological effects. Most triterpenes have low toxicity to normal cells, which makes them potentially safe for the body. Chemical modifications may additionally reduce toxicity and/or increase specificity of action on cancer cells. Using triterpenes creates proper opportunities for the synergistic effect of these compounds with other therapies (especially supporting anticancer therapies) and reducing side effects. In turn, the presence of triterpenes in numerous natural sources makes them easily available. For example, oleanolic acid, one of the most common triterpene acids, is isolated from more than 1600 species of plants [1] and certain fungi, such as *Ganoderma lucidum* [2] and *Inonotus obliquus* [3].

Triterpenes exhibit a wide range of chemical diversity. From a chemical point of view, this large group of natural compounds can be divided into several subgroups, with oleananes and lupanes as the most important and the most widely distributed groups. One of the most typical functional groups of all the known triterpenes is the hydroxyl group at the C-3 position. This hydroxyl group can be subjected to the reaction of ester synthesis, e.g., with the use of carboxylic acids [4], their anhydrides [5,6] or acyl chlorides [7]. Such transformations give, e.g., acetates [4,6], succinates [4], phthalates [5] or nicotinates [7] and other 3-*O*-esters.

Pharmacological tests have demonstrated various pharmacological activities of oleanolic acid, erythrodiol, allobetulin, betulinic acid, betulin and lupeol. Examples of pharmacological activity of the mentioned triterpenes are presented in Table 1. Table 1 also includes example results of antibacterial, antiviral and anticancer activity tests for the mentioned six triterpenes.

Cancer diseases, as well as many other diseases, e.g., autoimmune disorders, aging, cataracts, rheumatoid arthritis, cardiovascular and neurodegenerative diseases, are caused by, among other factors, oxidative stress [57]. It is caused by excessive production of Reactive Oxygen Species (ROS, free radicals) or their improper use by the body [58]. Under normal physiological conditions, free radicals benefit cellular responses and immune function [57]. They generate oxidative stress at high concentrations, a deleterious process that can damage cell structures, such as DNA, lipids and proteins [59]. Antioxidant and free radicals scavenging activity of triterpenes are the subject of many works, e.g., [60,61].

To improve the solubility of triterpenes in cytotoxic assay media, various modifications of triterpenes are performed and tested. Acetylation seems to be one such modification, as 3-*O*-acetyl derivatives are more soluble in organic solvents and those of polar character. By comparing the cytotoxic activities of betulinic acid derivatives with various acyl groups at the C-3 position, Ahmad et al. found that the cytotoxic potency may be dependent on the length of the alkyl chain on the acyl group at the above position [62].

The numerous data available in the scientific literature concern complicated derivatives of the mentioned triterpenes, obtained due to multi-stage, complicated chemical transformations. The basic hypothesis in our research was the answer to the question: does acylation of triterpenes with selected acylating agents improve their ADMETox (absorption, distribution, metabolism, excretion and toxicity) profile and specific effect on cancer cells? We decided to perform the acylation reaction of compounds **1a**–**6a** to test the cytotoxic and antioxidant activity of basic and acylated triterpenes (**1a**–**6a** and **1b**–**6b**, respectively), to calculate the ADMETox parameters and to conduct molecular docking for the most active derivatives. In this way, we have expanded the data library regarding the pharmacological activity of six basic triterpenes (**1a**–**6a**) and their simple derivatives (**1b**–**6b**). Thus, our work brings many novelties to the body of data regarding the diverse properties of the six triterpenes mentioned (**1a**–**6a** and **1b**–**6b**).

While triterpenes such as oleanolic acid, betulin and their derivatives have been extensively studied for their anticancer and antioxidant properties, existing research predominantly focuses on complex, multi-step chemical modifications to enhance their bioactivity. In contrast, our study introduces a simplified yet innovative approach: the direct acetylation of six foundational triterpenes (**1a**–**6a**) using acetic anhydride without toxic pyridine as an acetylating agent. Furthermore, while prior works often explore isolated compounds or complex derivatives, our research provides the first systematic comparison of acetylated vs. non-acetylated triterpenes across six cancer cell lines, including understudied derivatives such as compounds **3b** and **6b**.

The urgency of this work lies in the critical need to optimize natural triterpenes—compounds with inherently low toxicity—into clinically viable agents. Despite their promise, poor solubility and variable efficacy limit their therapeutic application. We address these limitations by focusing on simple structural modifications while prioritizing safety and scalability, offering a pragmatic pathway to develop potent, selective anticancer candidates.

## 2. Results

### 2.1. Acylation of Triterpenes

**Figure 1:**
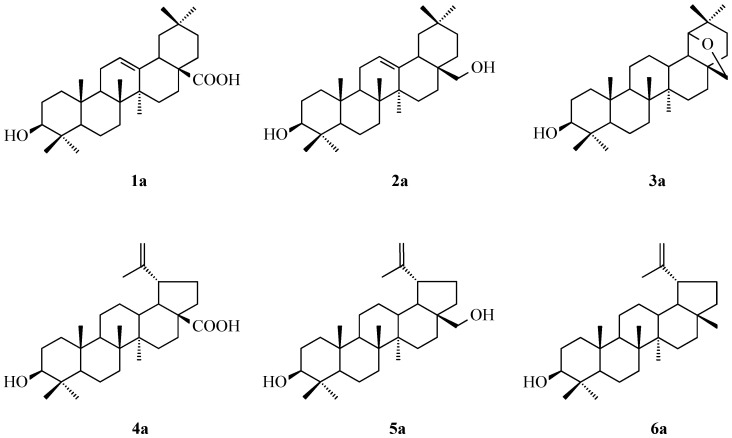
The structure of used triterpenes: oleanolic acid (**1a**), erythrodiol (**2a**), allobetulin (**3a**), betulinic acid (**4a**), betulin (**5a**) and lupeol (**6a**).

For our experiments, six triterpenes were applied: oleanolic acid (**1a**), erythrodiol (**2a**), allobetulin (**3a**), betulinic acid (**4a**), betulin (**5a**) and lupeol (**6a**) (Figure 1). Erythrodiol (**2a**) was obtained from oleanolic acid (**1a**) or its methyl ester (structure not given in Figure 1) via lithium aluminum hydride (LiAlH_4_) reduction. Allobetulin (**3a**) was synthesized from betulin based on a method from the literature [6].

**Figure 2:**
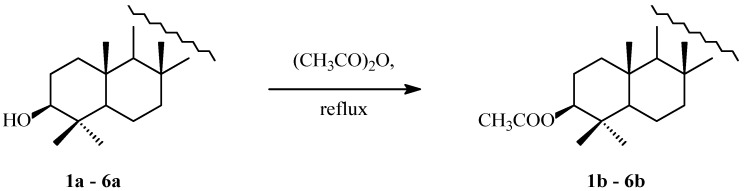
Transformation of triterpenes (**1a**–**6a**) into their acetyl derivatives (**1b**–**6b**).

The triterpenes **1a**–**6a** with the C-3 free OH group (Figure 1) were acylated in boiling acetic anhydride (Figure 2), without using toxic pyridine as a solvent. Spectral data of the isolated acylated triterpenes **1b**–**6b** are given in Supplementary Materials.

### 2.2. SAR Analysis

The highest results of structure–activity relationship (SAR) analysis unsubstituted and acetylated triterpenes (**1a**–**6a** and **1b**–**6b**) determined by the PASS method (prediction of activity spectra for substances) are given in Table 2.

### 2.3. In Vivo Assay

#### 2.3.1. MTT Results

The cytotoxic activity of **1a**–**6a** and **1b**–**6b** was tested with MTT (3-[4,5-dimethylthiazol-2-yl]-2,5 diphenyl tetrazolium bromide) assay for the following cancer cell lines: HeLa (*human cervical epithelioid carcinoma*), KB (*human oral squamous carcinoma*), MCF-7 (*human breast carcinoma*), A-549 (*human lung carcinoma*), PC-3 (*human prostate carcinoma*) and SKOV-3 (*human ovarian carcinoma*) and for comparison to normal cell line HDF (*human dermal fibroblasts*). The results are given as IC_50_ (half-maximal inhibitory concentration) and presented in Table 3.

#### 2.3.2. Selectivity Index

The selectivity index (SI) values for triterpenes **1a**–**6a** and their acetyl derivatives **1b**–**6b**, determined in the MTT assay, are given in Table 4.

#### 2.3.3. The Apoptosis Assay

The apoptotic index (AI) was calculated for the four most active triterpenes (**1a**, **5a**, **1b**, **4b**) and one example of a weakly active triterpene (**3a**) and for two cell lines the most susceptible to the action of the tested triterpenes are presented in Table 5.

### 2.4. Molecular Docking

#### 2.4.1. Detecting Cavities

The CB-Dock web server searches concave surfaces for cavities (a method called CurPocket).

**Figure 3:**
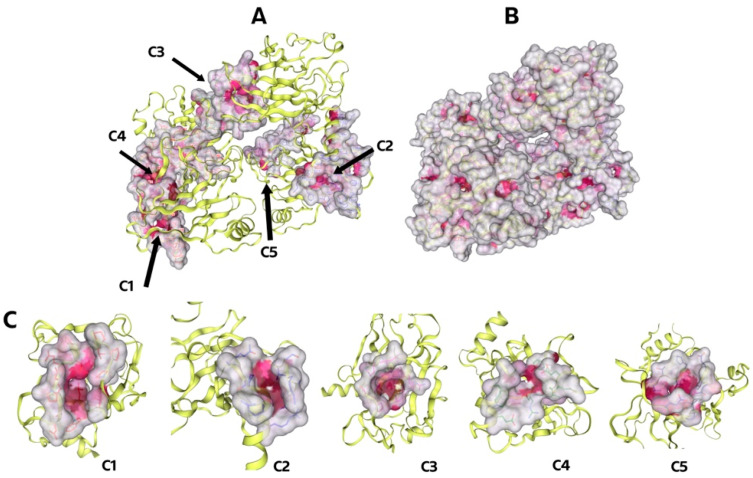
(**A**). Graphical result of searching the top 5 cavities for p53 Y220 (PBD ID: 8DC4), where the largest is C1 and the smallest is C5; (**B**). whole 8DC4 molecule, the dark pink color indicates the cavity of the molecule; (**C**). a different angle is shown to show the cavities better.

Below are the results for the crystal structure of p53 Y220 covalently bound to carbazole KG3 (PBD ID: 8DC4), the cavities of which are highlighted in Figure 3. We have selected the top five cavities as candidates for blind docking. The cavities were ranked by size, with the largest C1 and the smallest C5 (Table 6)**.**

#### 2.4.2. Molecular Docking

Results were obtained for 12 compounds, of which **1a**–**6a** contained free OH groups at the C3 atom in the molecule, and their acyl derivatives **1b**–**6b**. Based on the data presented in Table 7 and Table S2 (attached in Supplementary Materials), it can be concluded that the docking process occurs in all cavities (C1–C5) of the p53 Y220 protein molecule (PDB: 8DC4) and that all compounds tested show a good ability to interact with this structure. Details are shown in Table 7.

The highest result values were obtained for pocket C1, which is the largest, with a calculated volume of 3942 Å^3^; compound **5b** achieved the best docking result (−9.1 kcal × mol^−1^). Then, in pocket C2 with a volume of 2320 Å^3^, the highest result was achieved for compound **1b** (−9.0 kcal × mol^−1^). In pocket C3, compound **2b** showed the best fit, achieving a score of −10.1 kcal × mol^−1^, while in pocket C4, compound **6a** achieved the best result (−9.6 kcal × mol^−1^). For the C5 pocket, compound **4b** achieved a result of −9.0 kcal × mol^−1^.

**Figure 4:**
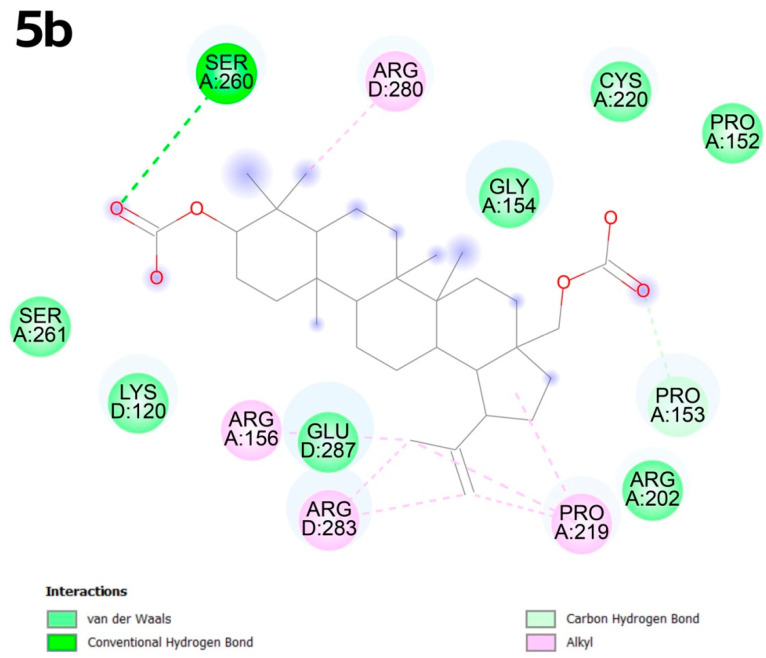
Docking score for compound **5b** with p53 Y220 (PBD ID: 8DC4), Vina score for pocket C1 is −9.1 kcal × mol^−1^.

Compound **5b** exhibits alkyl interactions with amino acids such as arginine at positions D:280, A:156 and D:283. The electro-negative oxygen atom in the carboxyl group promotes the formation of a conventional hydrogen bond with serine A:260, while the oxygen atom in the acyl group promotes the formation of van der Waals-type bonds (Figure 4).

**Figure 5:**
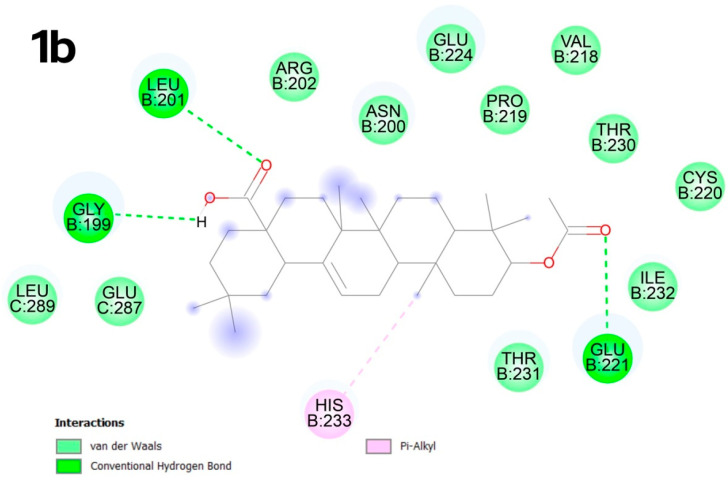
Docking score for compound **1b** with p53 Y220 (PBD ID: 8DC4). Vina score for pocket C2 is −9.0 kcal × mol^−1^.

In contrast, compound **1b** in the C2 pocket forms numerous van der Waals bonds with the surrounding amino acids, as well as conventional hydrogen bonds between the acyl group and the amino acids GLY B:199, LEU B:201 and between the oxygen atom in the carboxyl group and GLU B:221. An unsaturated bond promotes the formation of π-type alkyl interactions with histidine B:233 (Figure 5).

**Figure 6:**
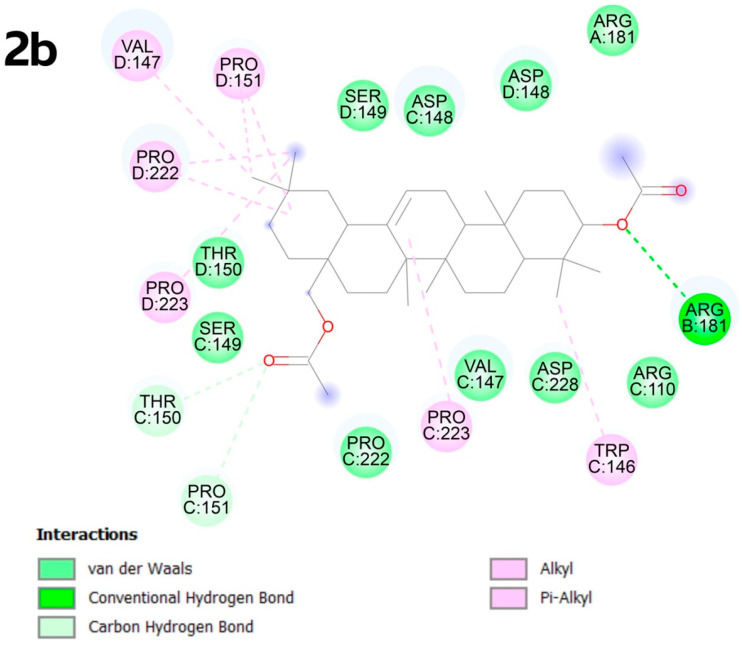
Docking score for compound **2b** with p53 Y220 (PBD ID: 8DC4). Vina score for pocket C3 is −9.3 kcal × mol^−1^.

In the C3 pocket, the best result was obtained for compound **2b**, which exhibits multiple alkyl bonds with proline amino acids at positions D:151, D:222, D:223, as well as with VAL D:147 and TRP C:146. Additionally, the unsaturated bond of molecule **2b** forms a π-type bond with PRO C:223. Carbon–hydrogen bonds occur due to the presence of oxygen atoms in the functional groups, forming interactions with THR C:150, PRO C:151 and ARG B:181 (Figure 6).

**Figure 7:**
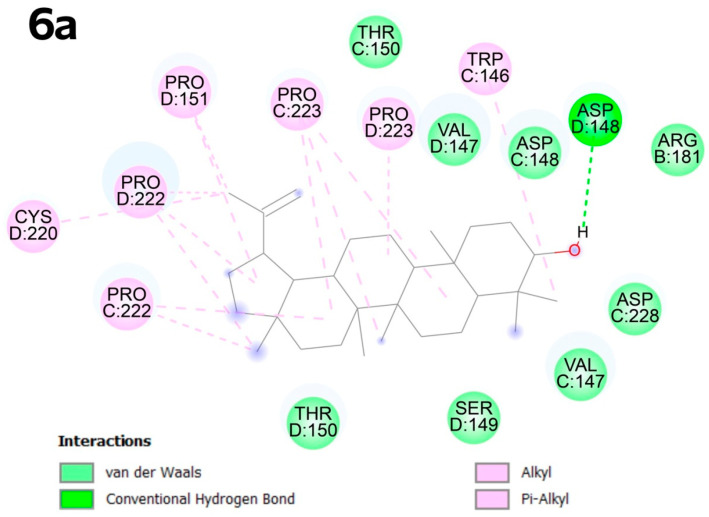
Docking score for compound **6a** with p53 Y220 (PBD ID: 8DC4). Vina score for pocket C4 is −9.6 kcal × mol^−1^.

In pocket C4, compound **6a** shows predominantly alkyl interactions with proline at positions D:151, C:222, D:222, D:223, and CYS D:220 and TRP C:146. The hydroxyl group at the C-3 atom favors the formation of a conventional hydrogen bond with ASP D:148 (Figure 7).

**Figure 8:**
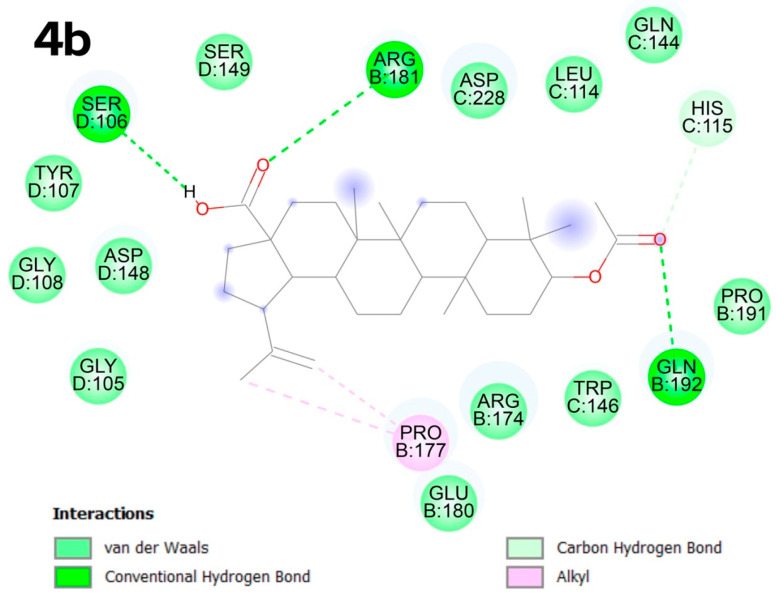
Docking score for compound **4b** with p53 Y220 (PBD ID: 8DC4). Vina score for pocket C5 is −9.0 kcal × mol^−1^.

In the C5 pocket, the best results were obtained for compound **4b**, in which van der Waals-type bonds predominate. The presence of oxygen atoms in the functional groups enables the formation of conventional hydrogen bonds with SER D:106, ARG B:181, GLN B:192, a carbon–hydrogen bond with HIS C:115 and an alkyl interaction with PRO B:177 (Figure 8).

### 2.5. Antioxidant Assay

**Figure 9:**
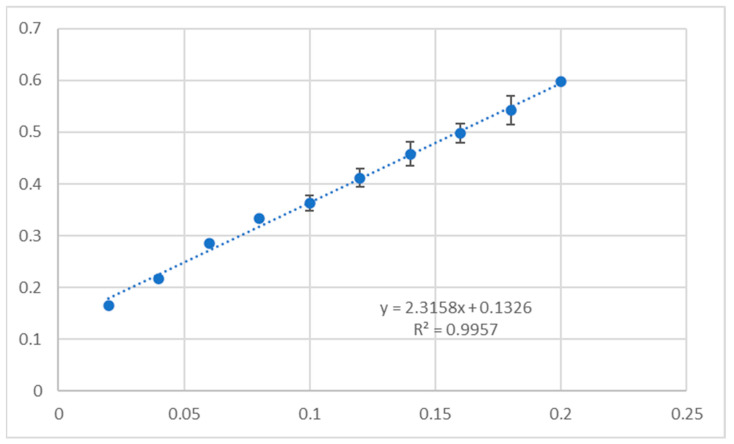
Standard curve for CUPRAC radical inhibition by Trolox.

**Figure 10:**
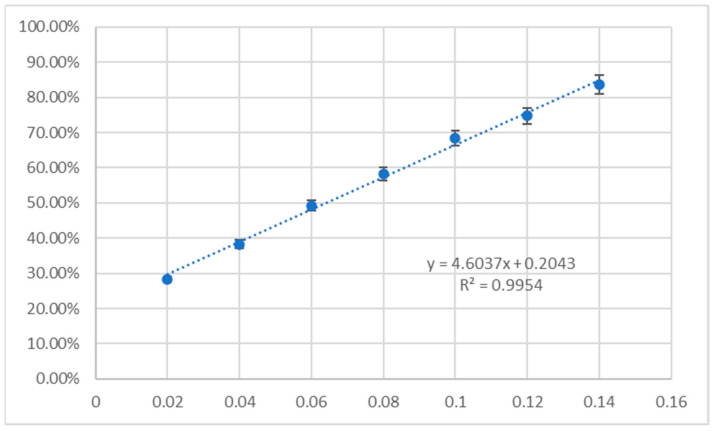
Standard curve for DPPH radical inhibition by Trolox.

The results of the antioxidant activity of unsubstituted triterpenes **1a**–**6a** and their acetylated derivatives **1b**–**6b** evaluated with CUPRAC (cupric reducing antioxidant capacity) and DPPH (2,2-diphenyl-1-picrylhydrazyl) assays are given in Table 8. The results are presented as % inhibition of the copper(II) ions and Trolox equivalent calculated from the standard curve (Figure 9) and as % inhibition of the DPPH radical and Trolox equivalent, calculated from the standard curve (Figure 10).

### 2.6. ADMETox Analysis

The physicochemical properties, pharmacokinetics and ADMETox (adsorption, distribution, metabolism, excretion and toxicity) activity of compounds **1a**–**6a** and **1b**–**6b** are given in Table 9.

Additional information concerning the above tests can be found in Supplementary Materials of our earlier publication [66].

## 3. Discussion

### 3.1. Acylation of Triterpenes ***1a**–**6a***

Acylation of the C-3 hydroxyl function of triterpene with carboxylic acid anhydrides gives the appropriate 3-*O*-acyl derivatives. Such a reaction is usually conducted using dried pyridine at room temperature [67] or in refluxed acetic anhydride [68]. In order to avoid using toxic pyridine, we decided to use the second of the methods we mentioned in another publication [68]. This reaction consisted of heating the triterpene in boiling acetic anhydride, and after the reaction was completed (TLC control), the reaction mixture was poured into water, and the separated white precipitate was filtered, washed with water, dried and crystallized.

### 3.2. Structure–Activity Analysis

Among the tested unsubstituted and acylated triterpenes (**1a**–**6a**, Figure 1, and **1b**–**6b**, respectively), most of the substances showed antineoplastic activity, with P_a_ values exceeding 0.900 for almost all tested compounds (**1a**–**6a** and **1b**–**6b**, Table 2). Allobetulin (**3a**) and its acetyl derivative (**3b**) showed the highest probability of high efficacy in the case of treatment of colon cancer (P_a_ > 0.900, Table 2) and only slightly lower in the case of treatment of lung cancer (P_a_ > 0.800).

The expected cytotoxic activity against cancer cells may be based on various mechanisms, e.g., enhancement of the process of apoptosis, stimulation of caspase 3 and caspase 8 or stimulation of TF NF kappa B/TF factor (Table 2).

Apoptosis, or programmed cell death, plays a vital role in cancer treatment by enabling the selective elimination of malignant cells while minimizing damage to surrounding healthy tissue. This highly regulated process is essential for maintaining cellular homeostasis and acts as a natural barrier against tumorigenesis. As a result, restoring the apoptotic machinery in cancer cells has become a central goal of modern oncology [69]. Substances that initiate or enhance this process play a key role in the apoptosis process. Such substances, called apoptosis agonists, activate the molecular pathways that lead to programmed cell death, restoring or intensifying the cell’s ability to undergo apoptosis when it is no longer viable or safe for the organism [69]. As shown by the prediction results using the PASS method (Table 2), oleanolic acid (**1a**) has the greatest chance of effective pro-apoptotic activity. Numerous publications in the scientific literature confirm these prediction results and demonstrate the proapoptotic effect of oleanolic acid (e.g., [70]).

Central to the execution of apoptosis is a family of proteolytic enzymes known as caspases (cysteine–aspartic proteases), which function as key molecular effectors of the apoptotic cascade. Caspases are generally classified into two groups: initiator caspases (e.g., caspase-8 and caspase-9), which respond to apoptotic signals and activate downstream pathways, and executioner caspases (e.g., caspase-3, -6 and -7), which carry out the dismantling of cellular components such as DNA, cytoskeletal proteins and nuclear structures. Activation of caspases can occur via two main apoptotic pathways: the extrinsic pathway, triggered by death receptors like Fas and TRAIL-R that activate caspase-8, and the intrinsic (mitochondrial) pathway, which involves mitochondrial outer membrane permeabilization and activation of caspase-9 via the apoptosome. Both pathways converge on executioner caspases, leading to systematic and non-inflammatory cell death. The essential role of caspases in apoptosis makes them critical targets for therapeutic intervention in diseases characterized by either excessive cell death (e.g., neurodegenerative diseases) or impaired apoptosis (e.g., cancers) [71,72,73].

Of the tested triterpenes **1a**–**6a** and **1b**–**6b**, the vast majority show a very high probability of caspase stimulant activity (P_a_ > 0.900, Table 2), in particular caspase 3 stimulant activity, with oleanolic acid again being the particularly active compound. This direction of pharmacological activity has also been confirmed in experimental studies and published [74].

Interestingly, betulinic acid (**4a**), as the only one among the twelve tested triterpenes (**1a**–**6a** and **1b**–**6b**), showed a moderate probability of caspase stimulant activity (Table 2). Betulinic acid (**4a**) has been widely studied for its anticancer potential, particularly its ability to induce apoptosis in various cancer cell types, e.g., [75,76]. However, its effectiveness as a direct caspase stimulant is limited. While triterpene **4a** can activate caspases downstream in the apoptotic cascade, especially caspase-3, it does so indirectly through mitochondrial dysfunction rather than direct activation of the caspase system. Specifically, betulinic acid primarily targets the intrinsic (mitochondrial) pathway, where it induces mitochondrial outer membrane permeabilization (MOMP), leading to cytochrome c release and apoptosome formation, which in turn activates caspase-9 and subsequently caspase-3 [75,76]. However, betulinic acid does not efficiently engage the extrinsic (receptor-mediated) pathway, nor does it directly bind or activate caspases themselves. Furthermore, its pro-apoptotic effects are often cell-type specific, and in some resistant tumor models, betulinic acid fails to induce strong caspase activation due to compensatory anti-apoptotic mechanisms such as upregulation of Bcl-2 or survival [77]. As a result, although betulinic acid is a promising compound for apoptosis induction, its role as a direct caspase stimulant is limited, and its efficacy may require combination with other agents that enhance or bypass caspase activation [77].

Nuclear factor kappa B (NF-κB) plays a key role in cancer by regulating inflammation, cell survival and proliferation genes. In many cancers, NF-κB is constitutively activated, enabling tumor cells to evade apoptosis through upregulation of anti-apoptotic genes like Bcl-2 and IAPs [78,79]. It also promotes angiogenesis, metastasis and therapy resistance by inducing cytokines, growth factors and survival proteins. Moreover, NF-κB shapes the tumor microenvironment by promoting chronic inflammation and immune evasion [80]). These functions make NF-κB a critical target in cancer therapy, though its role in normal immune function complicates systemic inhibition. As PASS prediction revealed (Table 2), both unsubstituted triterpenes **1a**–**6a** (Figure 1) and their acetyl derivatives (**1b**–**6b**, Figure 2) showed a high probability of transcription factor (TF)-stimulating activity, in particular TF NF kappa B, with a P_a_ value exceeding 0.900 for the vast majority of compounds tested (**1a**–**6a** and **1b**–**6b,** Figure 1 and Figure 2).

Triterpenes are known to modulate the transcription factor NF-κB due to their ability to influence key signaling pathways involved in inflammation and immune responses [81]. Many triterpenes, such as betulinic acid and oleanolic acid, can either activate or inhibit NF-κB depending on the cellular context and structure of the compound. Their lipophilic nature allows them to interact with membrane-bound receptors or intracellular signaling proteins like IKK (IκB kinase), influencing the degradation of IκB and subsequent translocation of NF-κB to the nucleus [81]. In some cancer or immune cells, triterpenes can stimulate NF-κB activity, potentially enhancing immune surveillance or inflammatory responses, while in other contexts they may inhibit chronic inflammation by blocking NF-κB activation [81]. This dual capacity makes them valuable pharmacological tools for modulating NF-κB signaling in both cancer and inflammatory diseases.

High P_a_ (probability of activity) values predicted by the PASS software for antitumor activity indicated a strong likelihood that the tested compounds possess cancer-inhibiting properties. This computational evidence supported the rationale for selecting the mutant p53 Y220C protein as a molecular target, as it plays a key role in tumor suppression and is frequently mutated in cancers. Targeting p53 Y220C aligns with the predicted bioactivity of the compounds, offering a focused approach to exploring their potential as reactivators or stabilizers of this dysfunctional tumor suppressor [82].

### 3.3. Cytotoxic Activity of Triterpenes ***1a**–**6a*** and ***1b**–**6b***

#### 3.3.1. In Vivo Assay

Six triterpenes with free hydroxyl function at the C-3 position, oleanolic acid (**1a**), erythrodiol (**2a**), allobetulin (**3a**), betulinic acid (**4a**), betulin (**5a**) and lupeol (**6a**) and their 3-*O*-acetyl derivatives (**1b**–**6b**, respectively), were subjected to the MTT assay. Four of these compounds have a carboxyl function at the C-17 position (**1a**, **4a**, **1b**, and **4b**), four triterpenes had a primary hydroxyl group at the C-17 position (**2a**, **5a**, **2b**, and **5b**) and four compounds had other functionalities at the C-17 position (**3a**, **6a**, **3b** and **6b**).

Among the six triterpenes, **1a**–**6a** that were tested against six human cancer cell lines—HeLa (*cervical carcinoma*), KB (*nasopharynx carcinoma*), MCF-7 (*breast carcinoma*), A-549 (*lung carcinoma*), PC-3 (*prostate carcinoma*) and SKOV-3 (*ovarian carcinoma*)—the most active turned out to be oleanolic acid (**1a**), with IC_50_ values in the range of 8.79 µM (for A-549 cells) to 18.63 µM (for PC-3 cells), betulinic acid (**4a**), with IC_50_ values from 9.52 to 35.21 µM (for SKOV-3 and KB cells, respectively) and betulin (**5a**), with IC_50_ values from 6.22 to 19.74 µM (PC-3 and KB cells, respectively) (Table 3). Reduction of the C-17 group of oleanolic acid (**1a**) leading to primary hydroxyl (as in a molecule of erythrodiol, **2a**) caused the obtained compound to be 4.4–7.2 times less active for HeLa, KB, MCF-7 and A-459 cell lines than oleanolic acid **1a**. The transformation of betulin **5a** into allobetulin **3a** caused a worsening of anticancer activity of the triterpene **3a**, with the IC_50_ values from about 20 to 70 µM (Table 3).

Acetylation of erythrodiol (**2a**), allobetulin (**3a**), betulin (**5a**) and lupeol (**6a**) led to derivatives with low or very low levels of cytotoxic activity, with IC_50_ values mainly in a range from 40 to > 100 µM. At the same time, the acetylation of oleanolic acid (**1a**) and betulinic acid (**4b**) led to a significant improvement in cytotoxic activity. The IC_50_ value for 3-*O*-acetyloleanolic acid (**1b**) varied from 0.09 µM (for the SKOV-3 cell line) to 1.86 µM (for MCF-7 cells); for 3-*O*-acetylbetulinic acid (**4b**) the IC_50_ value varied from 0.93 µM (for PC-3 cells) to 1.62 µM (for HeLa cells) (Table 3).

According to the literature data [83], chemical compounds present good cytotoxicity for IC_50_ ≤ 10 μM and moderate cytotoxicity for 10 μM < IC_50_ ≤ 30 μM. Taking into account the above statements and the IC_50_ values for six unsubstituted triterpenes, **1a**–**6a,** and their acetyl derivatives, **1b**–**6b**, the compounds with very high cytotoxic activity are acetyloleanolic acid (**1b**) and acetylbetulinic acid (**4b**), while their parent compounds, i.e., oleanolic acid (**1a**) and betulinic acid (**4a**), as well as betulin (**5a**) can be classified as compounds of high or moderately high activity (depending on the cancer cell line these compounds act on).

#### 3.3.2. Selectivity Index

The selectivity index for a given cancer cell line is the quotient of the IC_50_ value of a normal cell line (e.g., HDF, as in our experiments) and the IC_50_ value of a cancer cell line. This quotient is an important indication in research on the anticancer activity of both extracts and other preparations from materials of natural origin, individual substances isolated from these extracts and substances of natural origin subjected to chemical modifications. Sometimes, the SI value is a factor that determines whether tests of the cytotoxic activity of a preparation or substance will be continued. As Pena-Moran [84] states, the limit value of the selectivity index determining the validity of further research is at least 10. According to Valderrama, for individual substances that would become potential anticancer agents, the selectivity index limit value is at least 2 [85].

As Table 4 shows, the best selectivity index, approximately 2, for almost all tested triterpenes was obtained for two cancer cell lines: PC-3 and SKOV-3. Among all tested triterpenes, triterpene **1a** (oleanolic acid) and **4b** (diacetylbetulin) showed the highest results. For the first of the mentioned triterpenes (**1a**), the SI for the two cancer cell lines most susceptible to the action of triterpenes (PC-3 and SKOV-3) was much lower than for the remaining lines (HeLa, KB, MCF-7 and A-549) and was slightly above 1 (1.33 and 1.32, respectively; Table 4). In turn, the second triterpene with the most favorable selectivity index, i.e., diacetylbetulin (**4b**), showed the highest selectivity index value of approximately 3 for the PC-3 and SKOV-3 lines (Table 4).

#### 3.3.3. Apoptosis

The ability of all tested compounds to induce apoptosis was investigated with two cancer cell lines (SKOV-3 and PC-3). All twelve compounds (**1a**–**6a** and **1b**–**6b**) induced apoptosis to a similar extent in both cancer cell lines. Betulin (**5a**) was the most effective in inducing apoptosis as an internal standard. The lower concentration of 0.1 µg/mL showed a mean apoptotic index of 5.54 (Table 5). Oleanolic acid (**1a**), the second internal standard for investigated triterpenoids, showed an apoptotic index of 5.16 and 5.27, respectively, for the SKOV-3 and PC-3 cell lines (Table 5).

For other compounds, it was found that increased concentration also increased apoptotic indices. The investigation has shown that compounds **1b** (acetyloleanolic acid) and **4b** (acetylbetulinic acid) were the most effective in inducing apoptosis.

### 3.4. Molecular Docking

CB-Dock2 is an advanced molecular docking program distinguished by its high accuracy and efficiency in predicting binding positions and affinities between small molecules and target proteins. By considering ligand and protein flexibility and using a multiple protein conformation approach, CB-Dock2 better represents dynamic interactions than other rigid docking models. The program also uses advanced scoring functions and optimization algorithms, significantly improving predictions’ accuracy. CB-Dock2’s effectiveness has been repeatedly validated, making it a valuable tool in drug discovery and development [86,87,88].

The CB-Dock2 is not just a rough binding site determination tool but an advanced structure-based docking platform that also uses machine learning elements. It was designed as a blind docking method that integrates several docking approaches, including classical structural algorithms based on the detection of caverns on the protein surface and template-assisted docking of homologous protein–ligand complexes. This combination of structural and template algorithms allows CB-Dock2 to identify the ligand binding site precisely. According to the literature, the improved CB-Dock2 algorithm achieves about 85% accuracy in predicting the correct ligand binding pose (RMSD < 2.0 Å) in validation tests, outperforming the original CB-Dock and other popular blind docking tools. This means that CB-Dock2 provides accuracy comparable to traditional docking platforms and has internal validation mechanisms (e.g., via optional matching to homologous structures) that increase the results’ reliability [89].

In order to translate the PASS results into a specific molecular target, we considered proteins central to the regulation of cancer cell survival. Ultimately, we selected the p53 tumor suppressor protein (mutant Y220C) as a docking target, as its reactivation/stabilization is a known pro-apoptotic mechanism in cancer cells. The p53 Y220C variant has an allosteric pocket to which small molecules can bind to restore the function of this protein—making it an attractive target in the context of PASS indications with anticancer potential.

As a validation step, we redocked the co-crystallized ligand carbazole KG3 into the p53 Y220C structure (PDB ID: 8DC4]) using AutoDock Vina (version 1.2.0) integrated within the CB-Dock2 framework. This validation approach is consistent with standard docking protocols, where redocking of known ligands helps confirm the accuracy and reliability of the docking parameters [90,91]. Comparative analysis of binding energies relative to the co-crystallized ligand allowed for identifying compounds with potential to stabilize the p53 Y220C mutant conformation and restore its tumor suppressor function [92].

The tumor suppressor protein p53, which has long been the subject of intensive research, was chosen for docking, with its different isoforms and interactions playing a key role in cellular processes and disease pathogenesis. The p53 Y220 protein is a particular variant and has attracted considerable attention due to its potential in molecular docking applications. The p53 Y220 is a mutated form of the p53 protein found in the Protein Data Bank under ID 8DC4. This specific mutation has been linked to several oncogenic properties, including the ability to confer chemo-resistance and promote the growth and spread of malignant tumors [93,94].

A crystal structure study of p53 Y220, covalently bound to carbazole KG3 (PDB ID: 8DC4), was carried out to identify potential pockets on the protein’s surface, using the CB-Dock2 server. This enabled molecular docking to assess the interactions of compounds **1a**–**6a** and **1b**–**6b** with these pockets.

Using the CurPocket method, the CB-Dock2 server extracted five key pockets in the 8DC4 structure. The pockets were ordered by volume, from largest (C1) to smallest (C5). The largest pocket (C1) had a volume of 3942 Å^3^, while the smallest (C5) had a volume of 370 Å^3^. Details of the location and size of these pockets are shown in Table 7 and Figure 3.

Docking into multiple cavities (C1–C5) was conducted to identify each compound’s most favorable binding sites, as p53 Y220 is a mutant with altered surface topology. While energies are provided for all pockets, biological interpretation focuses on the most energetically favorable interactions—e.g., compound **2b** in pocket C3 (−10.1 kcal × mol^−1^), likely corresponding to the KG3 binding region. This approach ensures comprehensive exploration and prevents oversight of high-affinity noncanonical sites.

Molecular docking analysis showed that all compounds tested (**1a**–**6b**) could bind to each of the pockets (C1–C5), achieving different Vina score values, which reflect the strength of the ligand–protein interaction. The highest binding value was observed for compound **5b** in the C1 pocket, achieving a Vina score of −9.1 kcal × mol^−1^. Another favorable interaction was shown by compound **1b** in the C2 pocket with a value of −9.0 kcal × mol^−1^. In the C3 pocket, the highest Vina score value was achieved by compound **2b** (−10.1 kcal × mol^−1^), while for the C4 pocket the best result was obtained for compound **6a** (−9.6 kcal × mol^−1^). In the last pocket C5, the highest Vina score value was achieved by compound **4b**, with a result of −9.0 kcal × mol^−1^.

A lower affinity value (Vina score) indicates a stronger binding interaction between the molecules, indicating that all compounds tested showed good docking results and could inhibit interactions between the 8DC4 protein and cellular proteins. In the largest pocket, C1, the highest binding value was achieved for compound **5b**, while compound **1b** stood out in the C2 pocket, compound **6a** in the C4 pocket and compound **4b** in the smallest pocket, C5.

Molecular docking to the pockets identified on the surface of the p53 protein revealed various strong interactions between the compounds tested and the protein, suggesting that some of these compounds may represent potential inhibitors of p53. The best result was achieved by compound **2b** in the C3 pocket, which is about 4.8 times smaller than the largest pocket (C1).

Although larger pockets on the surface of the protein may offer more stable and potent binding sites for ligands, appropriate modification of the molecule—in this case, the addition of two acyl groups—gave better results than the variant with free hydroxyl groups. This result may suggest that the pocket prefers alkyl interactions, increasing binding stability.

### 3.5. Antioxidant Activity

The antioxidant activity results for the tested triterpenes **1a**–**6a** and **1b**–**6b**, measured using the CUPRAC and DPPH assays (Table 8), reveal significant differences in the antioxidant mechanisms of individual compounds. Analyzing the data for derivatives **1a**–**6a** and **1b**–**6b**, we can observe substantial variability in activity across both assays, reflecting the diverse antioxidant capacities of these compounds due to their structural properties and mechanisms of action.

The CUPRAC assay results show that compounds such as **5a**, **2b** and **1b** exhibit relatively high reducing activity (0.29900, 0.24016 and 0.21986, respectively; Table 8), suggesting that they have an efficient electron transfer capacity, which is a crucial factor in this assay. On the other hand, derivatives like **3b** and **3a** show significantly lower activity (0.03212 and 0.08248, respectively; Table 8), indicating a reduced ability to participate in redox reactions involving the reduction of copper(II) ions to copper(I). In contrast, the DPPH assay, which measures the ability of compounds to donate hydrogen atoms to neutralize free radicals, shows a different activity profile. For instance, derivative **5a**, which displayed the highest activity in the CUPRAC assay (0.29900; Table 8), exhibited one of the lowest activities in the DPPH assay (0.01077; Table 8). This suggests that the antioxidant mechanism of compound **5a** is primarily based on electron transfer rather than hydrogen atom donation.

Meanwhile, compound **2a** demonstrated relatively similar, albeit lower, activity in both assays (0.09414 in CUPRAC and 0.02092 in DPPH; Table 8), indicating a more versatile antioxidant mechanism encompassing electron transfer and hydrogen atom donation. These results underscore the crucial influence of a compound’s structure on its antioxidant properties. For example, derivative **6a**, which scored 0.13602 in the CUPRAC assay (Table 8), exhibited moderate activity in the DPPH assay (0.01570; Table 8), suggesting it effectively participates in both antioxidant mechanisms. Conversely, compounds such as **3b**, which showed the lowest activity in both assays (0.03212 in CUPRAC and 0.00182 in DPPH; Table 8), have limited antioxidant capacity, possibly due to an unfavorable structural configuration that impedes electron transfer and hydrogen atom donation. Further analysis of derivatives **1b**, **2b** and **4b**, which scored relatively high in the CUPRAC assay (0.21986, 0.24016 and 0.19986, respectively; Table 8) but much lower in the DPPH assay, highlights the importance of electron transfer as the dominant antioxidant mechanism for these compounds. Their intense activity in the CUPRAC assay suggests the presence of functional groups that favor redox reactions, which are less involved in hydrogen atom transfer mechanisms, explaining their lower DPPH activity—meanwhile, compounds **5b** and **5a** present an intriguing contrast. While **5a** showed the highest CUPRAC activity (0.29900; Table 8), it had relatively low DPPH activity (0.01077; Table 8). In contrast, **5b**, although demonstrating lower CUPRAC activity (0.17609; Table 8), displayed a significantly higher DPPH result (0.00786; Table 8), suggesting structural differences that influence their antioxidant mechanisms differently in the two assays.

In conclusion, the results indicate that the tested triterpenes exhibit varied antioxidant properties depending on the evaluation method. In particular, derivatives with high CUPRAC activity, such as **1b**, **2b** and **4b**, may have promising potential for therapeutic applications in oxidative stress-related conditions that require strong reducing properties.

### 3.6. ADMETox Analysis

Among the tested compounds, unsubstituted and acetylated triterpenes (**1a**–**6a** and **1b**–**6b**, respectively), most of the above-mentioned triterpenes had a favorable molecular weight and also showed favorable values of most parameters determining the physicochemical properties, e.g., nHA, nHD, nRot, nRing, nHet, fChar, nRig, flexibility and TPSA (Table 9). Due to the very low solubility of the above compounds in water, the values of logP and logD were outside the optimal range (Table 9).

The QED (quantitative estimation of drug-likeness) test showed that all the tested compounds (**1a**–**6a** and **1b**–**6b**) are similar to known drugs (QED ≥ 0.67). The number of sp^3^ hybridized carbons in the above 12 triterpenes is ≥0.42, which is a favorable value. The MCF18 value for all tested triterpenes (**1a**–**6a** and **1b**–**6b**, respectively) exceeds 45, which means a high level of novelty, following the trends currently observed in medicinal chemistry. In turn, the NP value (natural product-likeness, Npscore) within the range of 3.0–3.3 (Table 9) confirms the high similarity to compounds of natural origin (from which compounds **1b**–**6b** were obtained).

PAINS, BMS and Chelator tests are negative for almost all tested triterpenes, which means that there are no unfavorable elements of the structure of the molecules of these substances, which can be potentially responsible for toxicity or may, for example, enter into chemical interaction with other chemical substances present in the body (Table 9).

The Caco-2 test showed excellent or very good permeability of all of the tested triterpenes (**1a**–**6a** and **1b**–**6b**). The excellent or outstanding absorption and permeation profile has been confirmed by subsequent ADMET parameters, e.g., HIA, F_20%_ (Table 9).

Theoretical predictions indicate that almost all tested triterpenes (**1a**–**6a** and **1b**–**6b**) will probably bind well to plasma proteins and perfectly penetrate the blood-brain barrier, showing excellent volume distribution (about 1 L × kg^−1^) and proper percentage of the fraction unbound to plasma proteins (Table 9).

The excretion of the tested triterpenes is predicted by applying CL and T_1/2_ tests. The clearance of a drug (CL) is an important pharmacokinetic parameter that defines, together with the volume of distribution, the half-life and thus the frequency of dosing of a drug. The clearance of the tested triterpenes (**1a**–**6a** and **1b**–**6b**) was in a range of 2–6 mL × min^−1^ × kg^−1^ (apart from triterpene **2a**, with CL about 10.5 mL × min^−1^ × kg^−1^), with a high probability of being short half-life compounds (T_1/2_ ≤ 0.300) (Table 9).

Almost all triterpenes showed a very low probability of toxicity and biotoxicity. This means that these compounds can be used as non-toxic drugs (Table 9).

## 4. Materials and Methods

### 4.1. NMR

The ^1^H- and ^13^C-NMR spectra of compounds **1a**–**6a** and **1b**–**6b** were recorded using a Bruker Advance 600 MHz spectrometer (Billerica, MA, USA) with CDCl_3_ as a solvent, with tetramethylsilane (TMS) as an internal standard (Sigma-Aldrich^®^, Darmstadt, Germany). Chemical shifts (δ) were expressed in parts per million (ppm) relative to tetramethylsilane (TMS) as an internal standard, using CDCl_3_ as a solvent. Coupling constants (*J*) were expressed in Hertz (Hz).

### 4.2. Acetylation of Triterpenes ***1a**–**6a***

#### 4.2.1. General Information

Oleanolic acid (**1a**) was purchased from Bio-Tech^®^, Beijing (China). Erythrodiol (**2a**) and allobetulin (**3a**) were synthetized from oleanolic acid (**1a**) and betulin (**5a**), respectively. Betulinic acid (**4a**), betulin (**5a**) and lupeol (**6a**) were purchased from Natchem^®^ (Kraków, Poland). The purity of the substrates and the obtained products was confirmed by HP TLC and NMR methods.

All commercially available solvents and reagents used in our experiments were graded “pure for analysis” (Sigma-Aldrich^®^, Darmstadt Germany; Fluka^®^, Charlotte, NC, USA; Chempur^®^, Piekary Śląskie, Poland; and POCh^®^, Gliwice, Poland). The solvents were dried according to the usual procedures.

The melting points were measured with the Büchi apparatus in an open capillary and are uncorrected.

#### 4.2.2. Syntheses

**Oleanolic acid or methyl oleanolate reduction with LiAlH_4_**: A solution of oleanolic acid (**1a**) or its methyl ester (1 mmol) in 5 mL of dried THF was added dropwise to a suspension of LiAlH_4_ (10 mmol) in 5 mL of THF, and the resulting mixture was stirred for 1 h. Next, EtOH was added dropwise, and the mixture was filtered off and evaporated until dry. The obtained white solid was crystallized from EtOH. Yield: 93.0%, m.p. 230–232 °C (lit. m.p. 232.5–234 °C [95]).

**General procedure of triterpene acylation**: A solution of 1 mmol of triterpene **1a**–**6a** in 5 mL of dried acetic anhydride was refluxed for 30 min. After cooling, the mixture was poured into the 5-fold volume of water, and the resulting precipitate was filtered off, washed with water, dried and crystallized.

- 3-*O*-Acetyloleanolic acid (**1b**): m.p. 267–268 °C (lit. m.p. 268–269 °C [96]).
- 3,28-di-*O*-Acetylerythrodiol (**2b**): m.p. 185–187 °C (lit. m.p. 185–185.5 °C [95]).
- 3-*O*-Acetylallobetulin (**3b**): m.p. 285–285 °C (lit. m.p. 287–287.5 °C [97], lit. m.p. 280–281 °C [98]).
- 3-*O*-Acetylbetulinic acid (**4b**): m.p. 290–291 °C (lit. m.p. 285–290 °C [99]).
- 3,28-di-*O*-Acetylbetulin (**5b**): m.p. 215–217 °C (lit. m.p. 213–218 °C [98]).
- 3-*O*-Acetylolupeol (**6b**): m.p. 216–218 °C (lit. m.p. 217–218 °C [98]).

### 4.3. SAR Analysis

The structure–activity relationship analysis was performed using the PASS (prediction of activity spectra for substances) computer system [100]. It is a program that predicts many types of pharmacological activities and their mechanisms based on the structure of the analyzed chemical substance. Such mathematical analysis results in a list of probable pharmacological activities or their mechanisms. The probability of a given activity occurring (marked with the P_a_ symbol) or the non-occurrence of a given activity (marked with the P_i_ symbol) is assessed on a scale from 0.000 to 1.000.

### 4.4. MTT Assay

All the cell lines and mediums were obtained from the American Type Culture Collection (ATCC) supplied by LGC-Standards (Lomianki, Poland). Human cancer cell lines were cultured as follows: HeLa (*human cervix carcinoma*) and KB (*human oral squamous carcinoma*) lines were cultured in DMEM (Dulbecco’s Modified Eagle’s Medium); MCF-7 (*human breast carcinoma*) and A-549 (*human lung carcinoma*) were cultured in RPMI 1640 medium; PC-3 (*human prostate carcinoma*) was cultured in F-12K medium; SKOV-3 (*human ovarian cystadenocarcinoma*) was cultured in McCoy’s modified medium; HDF (*human normal dermal fibroblasts*) was cultured in Fibroblast Basal Medium. Each medium was supplemented with 10% fetal bovine serum, 1% L- glutamine and 1% penicillin/streptomycin solution. The cell lines were kept in an incubator at 37 °C.

All the tested cell lines (HeLa, KB, MCF-7, A-549, PC-3, SKOV-3 and HDF) were subjected to MTT assay. In short, this assay is based on the reaction between mitochondria enzymes (dehydrogenases) of tested cell lines and 3-(4,5-dimethylthiazol-2-yl)-2,5-diphenyltetrazolium bromide (MTT). The final product of this reaction gives violet formazan crystals. The number of live cells is proportional to the amount of formazan and is represented by the violet dye reagent. The details of the experiments are given in [19].

### 4.5. Apoptosis

The induction of apoptosis by tested compounds was determined with Cell Death Detection ELISA Plus from Roche Diagnostics GmbH (Mannheim, Germany).

Cell Death Detection ELISA Plus is an enzyme immunoassay for determining cytoplasmic histone-associated DNA fragments (mono- and oligonucleotides) after induced cell death. Cells (10^4^) were pipetted into wells of 96-well plates, and test compounds were added at final concentrations of 0.1, 1.0 and 10.0 µg × mL^−1^. Cells incubated in the absence of test compounds served as negative controls. After 4 h of incubation, cells were harvested by centrifugation of the plates for 10 min at 200× *g*. Supernatants were removed carefully, and cell pellets were resuspended in 200 µL of cell lysis buffer and incubated for 30 min at room temperature. After centrifugation of the plates (10 min at 200× *g*), 20 µL of cell lysates was transferred in duplicate into wells of provided streptavidin-coated microplates. Then, 80 µL of the immunoreagent containing a mixture of anti-histone-biotin monoclonal antibody and anti-DNA-peroxidase monoclonal antibody was added to each well. The microplates were covered with a provided adhesive foil and incubated for 2 h at room temperature. Thereafter, the solution in the wells was removed by tapping the microplate. The wells were rinsed three times with incubation buffer, and 100 µL of substrate (2,2′-azino-bis [3-ethylbenzothiazoline-6-sulphonic acid], ABTS) was added to each well. After 10–20 min, 100 µL of ABTS stop solution was pipetted to each well, and the absorbance was read at 405 nm (test wavelength) and 490 nm (reference wavelength). The apoptotic index was calculated based on the enrichment of mono- and oligonucleosomes according to the following equation:

apoptotic index = [{A_405(sample)_ − A_490(sample)_}/{A_405(negative control)_ − A_490(negative control)_}]

### 4.6. Molecular Docking

**Ligand preparation**: The ligands were prepared by first drawing the two-dimensional (2D) structures of compounds **1a**–**6a** and **1b**–**6b** using ChemDraw 22.0.0. These structures were then converted into three-dimensional (3D) representations in OpenBabel format [101] to obtain the coordinates representing the most energetically favorable conformation. Geometry optimization was performed using Avogadro version 1.2.0 software. The Steepest Descent algorithm applied the Universal Force Field (UFF). The optimized 3D structures of compounds **1a**–**6a** and **1b**–**6b** were saved as SDF files. They were used as input files for docking analysis on the CB-Dock2 server.

**Protein preparation**: The crystallographic data for the p53 protein (cancer mutation Y220C) with the 8DC4 structure was sourced from the RCSB Protein Data Bank (PDB ID: 8DC4) at a resolution of 2.40 Å. P53 is covalently bound to the carbazole KG3 in this X-ray crystal structure. The downloaded 8DC4.pdb protein molecule was not manually prepared, as steps such as removing ligands and crystal water molecules and adding missing hydrogen atoms were automatically handled by the CB-Dock2 server.

**Detecting cavities and uploading ligands**: After the p53 molecule was imported into the CB2-Dock server, the number of cavities for docking was set to 5 under the “more parameters” option. The email address to receive the output files was specified, and the Search Cavities function was selected. Ligand structures (compounds **1a**–**6a** and **1b**–**6b**) were uploaded to the CB-Dock2 server [101], and docking was initiated through the platform’s web interface using default parameters. The tool automatically identified the top five potential binding cavities based on cavity detection algorithms.

### 4.7. Antioxidant Activity

The CUPRAC reagent (7.5 mM ethanolic 96% neocuproine solution, 10 mM CuCl_2_ × H_2_O solution and an ammonium acetate buffer of pH 7.0) and DPPH reagent (2,2-diphenyl-1-picrylhydrazyl 0.2 mM solution) were applied.

The CUPRAC method measures the reducing power of a sample by quantifying its ability to convert the Cu-neocuproine reagent complex to the Cu(I) form. In contrast, the DPPH assay relies on the ability of an antioxidant to donate a hydrogen atom or an electron to the stable DPPH radical, resulting in its reduction and subsequent color change. The results are presented as % inhibition of the copper(II) ions and Trolox equivalent calculated from the standard curve (Figure 9) and as % inhibition of the DPPH radical and Trolox equivalent, calculated from the standard curve (Figure 10). The spectroscopic detection in CUPRAC and DPPH assays was conducted with Synergy H1 Multi-Mode Microplate Reader (BioTek Instruments, Inc., Winooski, VT, USA). The details of the experiments are given in [102].

### 4.8. ADMETox Profile

The physicochemical properties, pharmacokinetics and ADMETox (adsorption, distribution, metabolism, excretion and toxicity) activity of compounds **1a***–**6*****a** and **1b**–**6b** were estimated based on the comprehensive database ADMETlab Manual (2.0) [103]. First, the structures of the analyzed compounds were prepared using the JSME editor.

## 5. Conclusions

The conclusions from the research suggest that the acetylation of natural triterpenes, particularly oleanolic triterpenic acid and betulinic acid, significantly affects their cytotoxic and antioxidant properties. The most significant increase in activity was observed for acetyloleanolic acid (**1b**) and acetylbetulinic acid (**4b**), which showed very high cytotoxicity towards the tested cancer cell lines, with IC_50_ values in the micromolar or submicromolar range. This clearly exceeds the effect of their unmodified forms (**1a**, **4a**), suggesting that the modification at the C-3 position is crucial for anticancer activity.

Antioxidant tests using the CUPRAC and DPPH methods showed that acetylated triterpenes have different abilities to neutralize free radicals, which may result from differences in the mechanisms of action, depending on the chemical structure of the compounds. The results of ADMETox analyses also confirmed that most of the tested derivatives are characterized by a good pharmacokinetic profile and low toxicity, which makes them promising candidates for potential anticancer drugs. Docking results showed that all compounds tested (**1a**–**6a** and **1b**–**6b**) could bind to pockets (C1-C5) of the p53 Y220 protein, obtaining different Vina score values. The strongest binding was observed for compound **2b** in the C3 pocket (−10.1 kcal × mol^−1^), while in the largest C1 pocket, the best result was achieved by compound **5b** (−9.1 kcal × mol^−1^). The results suggest that larger pockets may offer more stable binding sites, but that appropriate structural modification, such as the presence of acyl groups, increases binding efficiency. The analysis indicates potential inhibitory properties of the selected compounds towards the p53 protein.

The acetylation reaction has essential functions. First of all, it is a simple reaction that allows for the reversible “blocking” of the hydroxyl (-OH), amino (-NH_2_) or thiol (-SH) group to prevent undesirable reactions. In addition, acetylation increases the molecule’s hydrophobicity, which can be beneficial when isolating or crystallizing compounds. The third reason for performing the acetylation reaction is to change the pharmacological activity of the molecules—chemically modified molecules can react in a more controlled, more selective way, or acetyl derivatives simply become more active than their unsubstituted parent compounds.

Table 10 compares anticancer activity between oleanolic acid and its acetylated derivative, acetyloleanolic acid.

The data in Table 10 clearly demonstrate that substituting the hydroxyl group (-OH) at the C-3 position of the oleanolic acid molecule with an acetoxy group (-OCOCH_3_) consistently results in a noticeable improvement in anticancer activity across various cell lines. This observation strongly supports the conclusion that acetylation of triterpenes is not merely a method for protecting or blocking the reactive hydroxyl group at C-3, but it also serves as a simple, cost-effective and highly efficient approach to enhancing the pharmacological properties of triterpenes—particularly their cytotoxic or anticancer potential. These findings reinforce the value of such chemical modifications in medicinal chemistry and drug development, especially in the search for more potent and selective anticancer agents.

To sum up, the obtained results suggest that the acetylation of natural triterpenes, especially those with carboxyl groups at the C-17 position, may lead to compounds with high biological activity. Further research on structural modifications and mechanisms of action of acetylated triterpenes is recommended to exploit their potential fully in anticancer therapy.

## 6. Future Research Directions

While some triterpenes (e.g., oleanolic acid, betulinic acid) have been studied, their acetylated derivatives’ comparative pharmacological profiles—especially against multiple cancer lines (HeLa, PC-3, SKOV-3) and their antioxidant mechanisms—are underexplored. Our work systematically evaluates these aspects, highlighting structure–activity trends that were not previously quantified (e.g., IC_50_ values for compounds **3b**, **5b**).

The presented work brings significant novelty in the following ways:

1. **First comparative evaluation:** Our study is the first to juxtapose acetylated and non-acetylated triterpenes across six cancer cell lines (HeLa, KB, MCF-7, A-549, PC-3, SKOV-3), including rarely investigated derivatives like **3b** and **6b**. This broad-spectrum analysis reveals stark contrasts in cytotoxicity, with acetylated derivatives **1b** and **4b** exhibiting submicromolar IC_50_ values, underscoring the transformative impact of C-3 acylation.
2. **Systematic acylation effects:** By systematically modifying six triterpenes, we identify structure–activity relationships (SARs) that highlight the critical role of the C-17 carboxyl group. For instance, acetylation of oleanolic acid (**1a**) and betulinic acid (**4a**) enhanced activity by 40–100-fold, whereas analogous modifications in erythrodiol (**2a**) or lupeol (**6a**) reduced efficacy, emphasizing the necessity of tailored functionalization.
3. **Mechanistic insights via novel docking:** Unlike prior studies, we explore interactions with the p53 Y220C mutant (PDB: 8DC4), a high-priority oncogenic target. Molecular docking using CB-Dock2—a machine learning–enhanced tool—revealed unique binding modes, such as **2b**’s strong affinity for the C3 pocket (−10.1 kcal × mol^−1^), suggesting potential inhibition of p53-driven tumorigenesis.
4. **CB-Dock2 advancements:** Our use of CB-Dock2, which incorporates ligand and protein flexibility, outperformed traditional rigid docking methods. This approach provided unprecedented accuracy in predicting binding poses, as evidenced by **5b**’s alkyl interactions with arginine residues in the C1 pocket, validating its utility in drug discovery.
5. **Integrated methodology:** By combining cytotoxicity, apoptosis, antioxidant assays and ADMETox profiling with computational analyses, we provide a holistic pharmacological evaluation. For example, **1b**’s high CUPRAC activity (0.21986 mg × mL^−1^ Trolox equivalent) contrasted with its low DPPH response, highlighting electron transfer as its primary antioxidant mechanism—a distinction critical for therapeutic applications.

Future directions of our research will focus on four aspects:

- **Structural modifications**: An example of planned chemical transformations is the introduction of additional functional groups (e.g., nitro, halogens) to increase molecular interactions with target proteins. More branched acyl groups will be introduced into the triterpene molecule to optimize lipophilicity and penetration of cell membranes.
- **Synergistic studies**: The combination of unsubstituted/acetylated triterpenes with other active substances (e.g., kinase inhibitors) may lead to synergistic anticancer activity.
- **Exploration of mechanisms of action**: the moderate activity of some triterpene derivatives may result from mechanisms of internal cellular resistance (e.g., expression of MDR pumps). Studying these mechanisms may indicate ways to improve pharmacological activity.
- **Application of nanocarriers**: Research on encapsulating compounds in lipid or polymer nanoparticles may increase their bioavailability and specificity towards cancer cells.

It is also worth expanding research on the antioxidant properties of these compounds, using other biological models, to better understand their potential in neutralizing oxidative stress associated with cancer. An essential aspect will also be optimizing the pharmacokinetic profile, including increasing the bioavailability and stability of compounds in the body, which may require further chemical modifications.

## Figures and Tables

**Table 1:** Examples of pharmacological activity of oleanolic acid, erythrodiol, allobetulin, betulinic acid, betulin and lupeol.

Triterpene	Examples of Pharmacological Activity	Ref.
**oleanolic acid**	antidiabetic	[8]
	neuroprotective	[9]
	hepatoprotective	[10]
	antioxidant	[11]
	antinociceptive and anti-inflammatory	[12]
	cardioprotective	[13]
	antihypertensive	[14]
	antiviral: EC_50_ = 13.07 µM (HSV-1/153), EC_50_ = 12.89 µM (HSV-1/106), EC_50_ = 13.09 µM (HSV-1/Blue)	[15]
	antibacterial: MBC = 700 µg × mL^−1^ (*Staphylococcus aureus*), MIC = 95 µg × mL^−1^ (*S. aureus*), MBC = 900 µg × mL^−1^ (*Escherichia coli*), MIC = 95 µg × mL^−1^ (*E. coli*), MBC = 300 µg × mL^−1^ (*Salmonella typhi*), MIC = 65 µg × mL^−1^ (*S. typhi*)	[16]
	antiatherosclerotic	[17]
	anticancer: IC_50_ = 14.93 µM (KB), IC_50_ = 13.95 µM (MCF-7), IC_50_ = 11.82 µM (HeLa), IC_50_ = 16.20 µM (Hep-G2)	[18,19]
	anticancer: IC_50_ = 40 µg × mL^−1^ (HCT-116)	[20]
	antioxidant, antiproliferative	[21]
**erythrodiol**	antioxidant, antiproliferative	[21]
	antioxidant, antiatherogenic	[22]
	Anti-inflammatory, vasorelaxing and cardioprotective	[23]
	antioxidant and anticancer: IC_50_ = 27.3 µM (Hep-G2)	[24]
	anticancer: EC_50_ = 48.8 µM (HT-29)	[25]
	anticancer: IC_50_ = 250 µM (CCRF-CEM, CEM)	[26]
	anticancer: IC_50_ = 64.96 µM (SMMC-7721), IC_50_ = 87.73 µM (Hep-G2), IC_50_ = 62.96 µM (A-459)	[27]
	antioxidant	[28]
**betulinic acid**	antidepressant	[29]
	antioxidant	[30]
	anxiolytic	[31]
	antiviral: EC_50_ = 11.2 μM (HCV)	[32]
	anti-inflammatory	[33]
	antiallergic and anti-inflammatory	[34]
	antihyperglycemic	[35]
	nephroprotective	[36]
	hepatoprotective	[37]
	anticancer: IC_50_ = 30 µM (CCRF-CEM, CEM)	[26]
	anticancer: IC_50_ = 125 µM (HT-29); IC_50_ = 58 µM (SW-480); IC_50_ = 178 µM (HCT-116)	[38]
	anticancer: IC_50_ = 30 µM (NOZ)	[39]
	anticancer: IC_50_ = 44.47 µM (A-2780)	[40]
**betulin**	anti-inflammatory	[41]
	wound healing	[42]
	antiviral: IC_50_ = 45.5 µM (SFV)	[43]
	antiseptic	[44]
	antihyperlipidemic	[45]
	antiobesity	[46]
	anticancer: IC_50_ = 250 µM (CCRF-CEM, CEM)	[26]
	anticancer: IC_50_ = 2.5 µM (SK-N-AS); IC_50_ = 5.9 µM (C-6); IC_50_ = 10.3 µM (TE-671); IC_50_ = 4.3 µM (HT-29); IC_50_ = 5.2 µM (T-47D); IC_50_ = 6.8 µM (FTC-238); IC_50_ = 7.4 µM (A-549); IC_50_ = 6.4 µM (RPMI-8226); IC_50_ = 6.7 µM (Jurkat 1E.6); IC_50_ = 2.8 µM (HPOC); IC_50_ = 3.4 µM (HPCC); IC_50_ = 3.4 µM (HPGBM)	[47]
	antioxidant; anticancer: IC_50_ = 29.4 µM (13 µg/mL; SGC7901)	[48]
	anticancer: IC_50_ = 73.2 µM (32.4 µg/mL; T-47D); IC_50_ = 24.6 µM (10.9 µg/mL; CCRF/CEM); IC_50_ = 51.7 µM (22.9 µg/mL; SW-707); IC_50_ = 12.4 µM (5.5 µg/mL; P-388)	[49]
**lupeol**	wound healing	[42]
	anti-inflammatory	[50]
	nephroprotective	[51]
	antiallergic	[52]
	antioxidant	[53]
	antiangiogenic	[54]
	anticancer: IC_50_ = 80 µM (MCF-7)	[55]
	anticancer: IC_50_ = 46.06 µM (MDA MB-231); IC_50_ = 31.910 µM (HeLa); IC_50_ = 64.82 µM (A-549)	[56]

**Legend**: **IC_50_** = half maximal inhibitory concentration; **EC_50_** = half maximal effective concentration; **BMC** = minimum bactericidal concentration; **MIC** = minimum inhibitory concentration; **HSV-1/153**, **HSV-1/106** and **HSV-1/Blue** = *Herpes simplex* virus type 1 ACV-resistant strains; **KB** = *human oral squamous carcinoma*; **MCF-7** = *human breast carcinoma*; **HeLa** = *human cervical epithelioid carcinoma*; **Hep-G2** = *human hepatocellular carcinoma*; **HCT-116** = *human colorectal carcinoma*; **HT-29** = *human colorectal adenocarcinoma*; **CCRF-CEM**, **CEM** = *human T lymphoblastic leukemia*; **SMMC-7721** = *human hepatocarcinoma*; **A-459** = *human lung carcinoma*; **HCV** = Hepatitis C virus; **SW-480** = *human colon adenocarcinoma*; **NOZ** = *human gall bladder carcinoma*; **A-2780** = *human ovarian carcinoma*; **SFV** = Semliki Forest virus; **SK-N-AS** = *human neuroblastoma*; **C-6** = *human glioma*; **TE-671** = *human rhabdomyosarcoma–medulloblastoma*; **HT-29** = *human colon adenocarcinoma*; **T-47D** = *human breast carcinoma*; **FTC-238** = *human thyroid carcinoma*; **RPMI-8226** = *human multiple myeloma*; **Jurkat 1E.6** = *human T cell leukemia*; **HPOC** = *human ovarian carcinoma*; **HPCC** = *human cervical carcinoma*; **HPGBM** = *human glioblastoma multiforme*; **SGC-7901** = human gastric carcinoma; **SW-707** = *human colon carcinoma*; **P-388** = *murine lymphocytic leukemia*; **MDA MB-231** = *human epithelial adenocarcinoma*.

**Table 2:** The PASS method calculated the predicted activity of unsubstituted and acetylated triterpenes (**1a**–**6a** and **1b**–**6b**, respectively).

Activity	P_a_ Factor (and P_i_ Factor) of Compounds 1a–6a and 1b–6b											
	1a	2a	3a	4a	5a	6a	1b	2b	3b	4b	5b	6b
**Antineoplastic**	0.876 (0.005)	0.920 (0.005)	**0.950** **(0.004)**	0.925 (0.005)	0.948 (0.004)	**0.950** **(0.004)**	0.890 (0.005)	0.923 (0.005)	**0.954** **(0.004)**	0.934 (0.004)	**0.952** **(0.004)**	**0.954** **(0.004)**
**Antineoplastic (colon c.)**	*<0.700*	0.734 (0.005)	0.917 (0.003)	0.789 (0.005)	0.853 (0.004)	0.831 (0.004)	*<0.700*	0.790 (0.005)	0.925 (0.003)	0.836 (0.004)	0.876 (0.004)	0.863 (0.004)
**Antineoplastic (colorectal c.)**	*<0.700*	0.736 (0.005	0.920 (0.003)	0.794 (0.005)	0.858 (0.004)	0.836 (0.004)	*<0.700*	0.791 (0.005)	0.927 (0.003)	0.840 (0.004)	0.879 (0.004)	0.867 (0.004)
**Antineoplastic (lung c.)**	0.766 (0.005)	0.802 (0.004)	0.883 (0.003)	0.815 (0.004)	0.833 (0.004)	0.850 (0.004)	0.792 (0.004)	0.823 (0.004)	0.899 (0.003)	0.831 (0.004)	0.859 (0.003)	0.869 (0.003)
**Antiprotozoal (Leishmania)**	0.721 (0.008)	*<0.700*	<*0.700*	0.923 (0.003)	0.881 (0.003)	0.891 (0.003)	0.821 (0.004)	0.848 (0.004)	0.790 (0.005)	**0.954** **(0.002)**	**0.961** **(0.002)**	0.940 (0.002)
**Apoptosis** **agonist**	0.901 (0.004)	0.892 (0.004)	0.759 (0.010)	0.822 (0.007)	0.837 (0.005)	0.883 (0.005)	0.891 (0.004)	0.878 (0.005)	0.747 (0.011)	0.850 (0.005)	0.825 (0.006)	0.874 (0.005)
**Caspase 3 stim.**	**0.984** **(0.002)**	**0.971** **(0.002)**	0.820 (0.005)	<*0.700*	**0.974** **(0.002)**	**0.978** **(0.002)**	**0.974** **(0.002)**	0.870 (0.004)	0.720 (0.010)	**0.976** **(0.002)**	0.880 (0.004)	**0.954** **(0.003)**
**Caspase 8 stim.**	0.914 (0.001)	0.878 (0.001)	0.808 (0.002)	<*0.700*	0.869 (0.001)	0.865 (0.001)	0.910 (0.001)	0.846 (0.001)	0.804 (0.002)	0.900 (0.001)	0.835 (0.001)	0.864 (0.001)
**Chemopre-** **ventive**	0.937 (0.002)	0.852 (0.003)	0.707 (0.006)	0.835 (0.003)	0.733 (0.005)	0.792 (0.004)	0.948 (0.002)	0.912 (0.002)	0.717 (0.006)	0.855 (0.003)	0.802 (0.004)	0.806 (0.004)
**Mucomem-** **branous prot.**	0.894 (0.005)	0.824 (0.013)	0.732 (0.042)	0.786 (0.022)	<*0.700*	0.847 (0.009)	0.935 (0.004)	0.892 (0.005)	0.795 (0.020)	<*0.700*	0.778 (0.025)	0.895 (0.005)
**Oxidoreductase inh.**	0.904 (0.002)	0.888 (0.003)	0.823 (0.005)	0.809 (0.006)	0.745 (0.010)	0.834 (0.005)	0.915 (0.002)	0.885 (0.003)	0.848 (0.004)	<*0.700*	0.724 (0.013)	0.855 (0.004)
**TF NF kappa B stim.**	**0.954** **(0.001)**	0.931 (0.001)	0.864 (0.002)	0.804 (0.003)	0.935 (0.001)	0.947 (0.001)	0.936 (0.001)	0.893 (0.002)	0.788 (0.003)	0.941 (0.001)	0.904 (0.001)	0.924 (0.001)
**TF stim.**	**0.954** **(0.001)**	0.931 (0.001)	0.864 (0.002)	0.804 (0.003)	0.935 (0.001)	0.947 (0.001)	0.936 (0.001)	0.893 (0.002)	0.788 (0.003)	0.941 (0.001)	0.904 (0.001)	0.924 (0.001)

**Legend**: **P_a_** = probability of activity, **P_i_** = probability of inactivity; **antag**. = antagonist, **inh**. = inhibitor, **prom**. = promoter, **prot**. = protectant, **stim**. = stimulant, **treatm**. = treatment; **c**. = cancer, **TF** = transcription factor.

**Table 3:** Results of MTT assay for unsubstituted and acetylated triterpenes (**1a**–**6a** and **1b**–**6b,** respectively) determined in the MTT assay.

Comp. No.	Cell Line, IC_50_ [µM] (±SD)						
	HeLa	KB	MCF-7	A-549	PC-3	SKOV-3	HDF
**1a**	11.82 (±0.19) *	14.93 (±0.07) *	13.95 (±0.11) *	8.79 (±0.20) *	18.63 (±0.05) ***	18.81 (±0.09) ***	24.87 (±0.04) ***
**2a**	54.32 (±0.02)	66.04 (±0.44)	65.52 (±0.18)	63.67 (±0.12)	21.09 (±0.18)	18.33 (±0.05)	27.99 (±0.66)
**3a**	49.44 (±0.21)	45.33 (±0.03)	71.06 (±0.13)	51.74 (±0.18)	20.17 (±0.04)	20.93 (±0.02)	48.22 (±0.19)
**4a**	27.50 (±0.12)	35.21 (±0.06)	27.89 (±0.12)	26.23 (±0.19)	10.61 (±0.09)	9.52 (±0.21)	19.92 (±0.49)
**5a**	18.32 (±0.02)	19.74 (±0.13)	18.09 (±0.02)	19.56 (±0.17)	6.22 (±0.06)	6.95 (±0.08)	15.81 (±0.66)
**6a**	37.74 (±0.12) **	51.17 (±1.92) **	51.82 (±0.15) **	45.70 (±0.12) **	14.52 (±0.03)	14.57 (±0.09)	29.04 (±0.06)
**1b**	**0.24** **(±0.19)**	**0.36** **(±0.07)**	**1.86** **(±0.16)**	**0.24** **(±0.12)**	**0.11** **(±0.02)**	**0.09** **(±0.01)**	**0.19** **(±0.07)**
**2b**	70.30 (±0.15)	49.96 (±0.31)	61.58 (±0.17)	51.61 (±0.43)	25.13 (±0.09)	25.51 (±0.04)	53.11 (±0.52)
**3b**	>100	>100	>100	>100	52.22 (±0.14)	53.09 (±0.55)	62.02 (±0.18)
**4b**	**1.62** **(±0.18)**	**1.50** **(±0.07)**	**1.50** **(±0.11)**	**1.23** **(±0.13)**	**0.93** **(±0.02)**	**1.03** **(±0.01)**	**2.88** **(±0.09)**
**5b**	>100	>100	>100	>100	75.91 (±0.37)	75.96 (±0.84)	81.73 (±0.49)
**6b**	72.64 (±0.14)	72.52 (±0.44)	49.97 (±0.31)	56.22 (±0.59)	22.16 (±0.15)	21.09 (±0.11)	34.97 (±0.39)

**Legend**: **HeLa** = *cervix carcinoma*; **KB** = *nasopharynx carcinoma*; **MCF-7** = *breast carcinoma*; **A-549** = *lung carcinoma*; **PC-3** = *prostate carcinoma*; **SKOV-3** = *ovarian carcinoma*; **HDF** = *normal dermal fibroblasts*; **±SD** = standard deviation; * = data published in [63]; ** = data published in [64]; *** = data published in [65].

**Table 4:** Selectivity index for unsubstituted and acetylated triterpenes (**1a**–**6a** and **1b**–**6b,** respectively) determined in the MTT assay.

Comp. No.	Cell Line, SI					
	HeLa	KB	MCF-7	A-549	PC-3	SKOV-3
**1a**	**2.10**	1.66	1.78	**2.83**	1.33 **	1.32 **
**2a**	0.87	0.42	0.43	0.44	1.32	1.53
**3a**	0.97	1.06	0.68	0.93	**2.39**	**2.30**
**4a**	0.72	0.56	0.71	0.76	1.88	**2.09**
**5a**	0.86	0.80	0.87	0.81	**2.54**	**2.27**
**6a**	0.77	0.57	0.56	0.63	**2.00**	1.99
**1b**	0.79	0.53	0.10	0.79	1.73	**2.11**
**2b**	0.75	1.06	0.86	1.03	**2.11**	**2.08**
**3b**	- - - *	- - - *	- - - *	- - - *	1.19	1.17
**4b**	1.77	1.92	1.92	**2.34**	**3.10**	**2.80**
**5b**	- - - *	- - - *	- - - *	- - - *	1.08	1.07
**6b**	0.48	0.48	0.70	0.62	1.58	1.66

**Legend**: **SI** = selectivity index; **HeLa** = *cervix carcinoma*; **KB** = *nasopharynx carcinoma*; **MCF-7** = *breast carcinoma*; **A-549** = *lung carcinoma*; **PC-3** = *prostate carcinoma*; **SKOV-3** = *ovarian carcinoma*; * = selectivity index was not calculated because the IC_50_ value exceeded 100 µM; ** = published in [65].

**Table 5:** Apoptotic index for the selected **1a**, **3a**, **5a**, **1b** and **4b** determined in the MTT assay for SKOV-3 and PC-3 cell lines.

Comp. No.	Cell Line, AI	
	SKOV-3	PC-3
**1a**	5.16 (0.01)	5.27 (0.04)
**3a**	5.78 (0.01)	5.27 (0.01)
**5a**	5.26 (0.03)	5.82 (0.03)
**1b**	6.79 (0.16)	6.90 (0.04)
**4b**	**7.81 (0.02)**	**7.09 (0.01)**

**Legend**: **AI** = Apoptosis index; **SKOV-3** = *ovarian carcinoma*; **PC-3** = *prostate carcinoma.*

**Table 6:** The results from the CB-Dock2 web server include the five largest cavities, from largest (C1) to smallest (C5), with their assigned calculated volumes (Å^3^), as well as the coordinates of their centers and dimensions expressed in angstroms (Å).

CurPocket ID	Cavity Volume (Å^3^)	Center (x, y, z)	Cavity Size (x, y, z)
**C1**	3942	−16, 49, 61	30, 18, 27
**C2**	2320	−6, 46, 9	18, 23, 18
**C3**	828	−33, 65, 34	11, 20, 8
**C4**	430	−40, 55, 40	19, 7, 8
**C5**	370	−30, 44, 24	8, 14, 15

**Table 7:** Docking results presented as Vina score values (kcal × mol^−1^).

Pocket ID	Compound Number											
	1a	1b	2a	2b	3a	3b	4a	4b	5a	5b	6a	6b
**C1**	−6.4	−9.0	−7.6	−7.1	−9.3	−8.3	−6.0	−7.8	−7.1	−9.1	−8.5	−9.1
**C2**	−6.3	−9.0	−6.5	−6.4	−6.5	−6.4	−6.2	−5.7	−5.8	−6.0	−6.0	−6.1
**C3**	−8.6	−7.4	−9.3	−10.1	−9.9	−9.3	−8.6	−9.3	−8.2	−8.1	−9.3	−9.6
**C4**	−8.2	−8.5	−8.0	−8.8	−9.4	−9.1	−8.6	−8.4	−8.0	−8.2	−9.6	−8.6
**C5**	−7.8	−7.8	−7.5	−8.9	−8.3	−7.9	−6.8	−9.0	−7.1	−7.5	−7.5	−7.2

**Table 8:** The results of the antioxidant activity of unsubstituted and acetylated triterpenes (**1a**–**6a** and **1b**–**6b,** respectively) evaluated with CUPRAC and DPPH assays.

Comp. No.	Trolox Equivalent [mg/mL]	
	CUPRAC Assay	DPPH Assay
**1a**	0.10312 ± 0.00684	**0.02633 ± 0.001090**
**2a**	0.09414 ± 0.00950	**0.02092 ± 0.000866**
**3a**	0.08248 ± 0.00500	0.01677 ± 0.000694
**4a**	0.07632 ± 0.00727	0.01526 ± 0.000632
**5a**	**0.29900 ± 0.00663**	0.01077 ± 0.000446
**6a**	0.13602 ± 0.00593	0.00065 ± 00.01570
**1b**	**0.21986 ± 0.00657**	0.01430 ± 0.000592
**2b**	**0.24016 ± 0.00995**	0.00313 ± 0.000130
**3b**	0.03212 ± 0.00166	0.00182 ± 0.000755
**4b**	0.19986 ± 0.00150	0.00376 ± 0.000156
**5b**	0.17609 ± 0.00487	0.00786 ± 0.000325
**6b**	0.13490 ± 0.00514	0.00348 ± 0.000144

**Table 9:** ADMETox data calculated for unsubstituted and acetylated triterpenes (**1a**–**6a** and **1b**–**6b**, respectively).

Properties (Optimal Values)	Compound Number											
	1a	2a	3a	4a	5a	6a	1b	2b	3b	4b	5b	6b
**Mol. Weight (100~600)**	456.360	442.380	442.380	456.360	442.380	426.390	498.370	526.400	484.390	498.370	526.400	468.400
**Volume**	505.750	499.598	493.678	505.751	499.598	490.807	546.497	581.089	534.423	546.497	581.089	531.553
**Density**	0.902	0.885	0.896	0.902	0.885	0.869	0.912	0.906	0.906	0.912	0.906	0.881
**NHA (0~12)**	3	2	2	3	2	1	4	4	3	4	4	2
**nHD (0~7)**	2	2	1	2	2	1	1	0	0	1	0	0
**nRot (0~11)**	1	1	0	2	2	1	3	5	2	4	6	3
**nRing (0~6)**	5	5	6	5	5	5	5	5	6	5	5	5
**maxRing (0~18)**	22	22	7	21	21	21	22	22	7	21	21	21
**nHet (1~15)**	3	2	2	3	2	1	4	4	3	4	4	2
**fChar (−4~+4)**	0	0	0	0	0	0	0	0	0	0	0	0
**nRig (0~30)**	27	26	29	27	26	26	28	28	30	28	28	27
**Flexibility (** **≤** **2)**	0.037	0.038	0.000	0.074	0.077	0.038	0.107	0.179	0.067	0.143	0.214	0.111
**Stereo Centers (** **≤** **2)**	8	8	10	10	10	10	8	8	10	10	10	10
**TPSA (0~140)**	57.530	40.460	29.460	57.530	40.460	20.230	63.600	52.600	35.530	63.600	52.600	26.300
**LogS (−4~0.5)**	−5.036	−5.769	−6.590	−5.148	−5.681	−6.667	−5.982	−6.964	−7.152	−5.866	−6.772	−7.189
**LogP (0~3)**	6.113	6.538	6.515	5.574	5.747	6.689	6.889	7.674	6.994	6.149	6.843	7.170
**LogD (1~3)**	4.843	4.705	5.145	4.873	4.682	5.313	4.873	4.975	5.214	4.945	4.971	5.417
**QED (** **≥** **0.67)**	0.409	0.424	0.428	0.436	0.452	0.421	0.311	0.274	0.356	0.320	0.274	0.299
**Sascore (** **≤** **6)**	4.589	4.702	5.524	4.689	4.761	4.663	4.624	4.754	5.542	4.725	4.823	4.692
**Fsp3 (** **≥** **0.420)**	0.900	0.933	1.000	0.900	0.933	0.933	0.875	0.882	0.969	0.875	0.882	0.906
**MCE-18 (** **≥** **45)**	105.368	102.207	116.200	104.000	100.793	100.793	106.667	105.000	117.397	105.300	103.594	102.164
**Npscore (−5~5)**	3.272	3.326	3.146	3.072	3.233	3.054	3.217	3.073	3.061	3.012	2.975	2.956
**Lipinski Rule**	A	A	A	A	A	A	A	R	A	A	R	A
**Pfizer Rule**	R	R	R	R	R	R	R	R	R	R	R	R
**GSK Rule**	R	R	R	R	R	R	R	R	R	R	R	R
**Golden Triangle**	A	A	R	A	A	R	A	R	R	A	R	R
**PAINS (alerts)**	0	0	0	0	0	0	0	0	0	0	0	0
**ALARM NMR (alerts)**	0	0	0	0	0	0	0	0	0	0	0	0
**BMS (alerts)**	0	0	0	0	0	0	0	0	0	0	0	0
**Chelator Rule (alerts)**	0	0	0	0	0	0	0	0	0	0	0	0
**Caco-2 perm. (≥** **−** **5.15)**	−5.198	−4.867	−5.115	−6.283	−4.942	−5.042	−5.166	−4.989	−5.040	−5.203	−5.029	−4.946
**MDCK perm.** **(≤2 × 10^−6^ cm/s)**	2.00 × 10^−5^	8.83 × 10^−6^	1.91 × 10^−5^	1.80 × 10^−5^	1.30 × 10^−5^	1.00 × 10^−5^	1.60 × 10^−5^	1.30 × 10^−5^	2.22 × 10^−5^	2.46 × 10^−5^	1.97 × 10^−5^	1.47 × 10^−5^
**Pgp-inh. (** **≤** **0.300)**	0.000	0.002	0.002	0.002	0.008	0.029	0.001	0.171	0.028	0.014	0.813	0.174
**Pgp-sub. (** **≤** **0.300)**	0.000	0.000	0.000	0.000	0.000	0.000	0.000	0.000	0.000	0.000	0.000	0.000
**HIA (** **≤** **0.300)**	0.012	0.011	0.005	0.007	0.004	0.003	0.014	0.019	0.005	0.007	0.005	0.004
**F_20%_ (** **≤** **0.300)**	0.074	0.878	0.250	0.340	0.880	0.742	0.031	0.455	0.018	0.012	0.021	0.014
**F_30%_ (** **≤** **0.300)**	0.756	0.952	0.956	0.898	0.939	0.891	0.828	0.929	0.865	0.899	0.962	0.833
**PPB (** **≤** **90%)**	98.130	98.230	97.480	96.515	97.762	98.998	97.013	97.247	96.056	96.841	95.967	98.268
**VD (0.04–20 L/kg)**	0.718	1.194	1.441	0.614	1.103	1.650	0.686	1.196	1.264	0.687	1.303	1.681
**BBB penetr. (** **≤** **0.300)**	0.674	0.562	0.376	0.492	0.471	0.627	0.329	0.254	0.176	0.206	0.262	0.353
**Fu (** **≥** **5.000%)**	3.524	2.365	2.072	2.579	1.625	1.713	3.461	1.927	2.151	2.123	1.714	1.646
**CYP1A2 inh. (** **≥** **0.700)**	0.012	0.038	0.032	0.021	0.047	0.037	0.011	0.022	0.028	0.016	0.029	0.033
**CYP1A2 sub. (** **≥** **0.700)**	0.323	0.194	0.336	0.549	0.333	0.598	0.166	0.119	0.152	0.243	0.130	0.455
**CYP2C19 inh. (** **≥** **0.700)**	0.028	0.054	0.063	0.027	0.054	0.073	0.029	0.070	0.068	0.026	0.065	0.080
**CYP2C19 sub. (** **≥** **0.700)**	0.916	0.924	0.932	0.928	0.939	0.947	0.893	0.914	0.931	0.931	0.930	0.948
**CYP2C9 inh. (** **≥** **0.700)**	0.157	0.187	0.100	0.129	0.142	0.093	0.218	0.196	0.101	0.138	0.125	0.090
**CYP2C9 sub. (** **≥** **0.700)**	0.813	0.124	0.175	0.703	0.160	0.569	0.723	0.080	0.143	0.678	0.110	0.596
**CYP2D6 inh. (** **≥** **0.700)**	0.012	0.049	0.050	0.005	0.031	0.057	0.012	0.111	0.033	0.005	0.079	0.029
**CYP2D6 sub. (** **≥** **0.700)**	0.528	0.245	0.837	0.764	0.807	0.898	0.177	0.096	0.675	0.600	0.475	0.879
**CYP3A4 inh. (** **≥** **0.700)**	0.172	0.717	0.252	0.127	0.519	0.223	0.246	0.605	0.278	0.155	0.530	0.250
**CYP3A4 sub. (** **≥** **0.700)**	0.208	0.455	0.335	0.223	0.464	0.433	0.377	0.667	0.504	0.325	0.580	0.525
**CL (** **≥** **15 mL/min/kg)**	3.094	10.509	4.701	3.039	6.486	5.372	2.119	3.091	3.454	2.454	3.165	3.552
**T1/2_< 3h_ (** **≤** **0.300)**	0.023	0.017	0.043	0.063	0.051	0.037	0.013	0.009	0.027	0.039	0.025	6.486
**hERG Blockers (** **≤** **0.300)**	0.004	0.021	0.511	0.031	0.056	0.124	0.003	0.008	0.326	0.021	0.044	0.102
**H-HT (** **≤** **0.300)**	0.296	0.402	0.254	0.275	0.262	0.085	0.278	0.313	0.271	0.298	0.258	0.126
**DILI (** **≤** **0.300)**	0.010	0.007	0.031	0.009	0.011	0.024	0.024	0.202	0.437	0.046	0.492	0.341
**AMES Tox. (** **≤** **0.300)**	0.008	0.003	0.005	0.003	0.002	0.002	0.005	0.002	0.003	0.002	0.002	0.002
**ROAT (** **≤** **0.300)**	0.228	0.162	0.168	0.221	0.129	0.185	0.080	0.021	0.038	0.045	0.009	0.032
**FDAMDD (** **≤** **0.300)**	0.909	0.925	0.939	0.928	0.928	0.919	0.623	0.753	0.727	0.628	0.726	0.515
**Skin Sensit. (** **≤** **0.300)**	0.028	0.076	0.893	0.329	0.624	0.727	0.036	0.035	0.532	0.195	0.129	0.680
**Carcinogen. (** **≤** **0.300)**	0.063	0.067	0.005	0.018	0.014	0.006	0.080	0.059	0.005	0.016	0.013	0.005
**Eye Corrosion (** **≤** **0.300)**	0.012	0.006	0.169	0.022	0.019	0.882	0.195	0.008	0.170	0.030	0.007	0.877
**Eye Irritation (** **≤** **0.300)**	0.084	0.022	0.263	0.039	0.047	0.550	0.050	0.036	0.196	0.032	0.068	0.458
**Respir. Tox. (** **≤** **0.300)**	0.968	0.980	0.934	0.945	0.841	0.580	0.968	0.958	0.802	0.928	0.603	0.412
**Bioconc. Factors**	1.944	3.075	2.476	2.117	2.841	2.560	2.422	2.784	2.323	2.553	2.444	2.410
**IGC_50_**	5.021	5.176	5.726	5.176	5.357	5.710	5.048	5.311	5.789	5.240	5.491	5.773
**LC_50_FM**	5.937	6.126	6.841	6.289	6.495	6.951	6.034	6.354	6.868	6.365	8.611	6.977
**LC_50_DM**	6.337	6.676	6.924	6.504	6.868	6.990	6.359	6.731	6.852	6.466	6.910	6.904
**NR-AR (** **≤** **0.300)**	0.369	0.055	0.002	0.069	0.021	0.007	0.665	0.167	0.019	0.459	0.075	0.116
**NR-AR-LBD (** **≤** **0.300)**	0.273	0.355	0.042	0.495	0.252	0.065	0.733	0.788	0.112	0.740	0.484	0.233
**NR-AhR (** **≤** **0.300)**	0.001	0.000	0.000	0.000	0.000	0.000	0.001	0.001	0.000	0.001	0.001	0.000
**NR-Aromatase (** **≤** **0.300)**	0.759	0.702	0.381	0.463	0.547	0.267	0.795	0.675	0.448	0.426	0.502	0.311
**NR-ER (** **≤** **0.300)**	0.412	0.249	0.309	0.115	0.116	0.272	0.721	0.178	0.286	0.135	0.069	0.229
**NR-ER-LBD (** **≤** **0.300)**	0.593	0.765	0.696	0.432	0.237	0.546	0.785	0.633	0.805	0.657	0.737	0.786
**NR-PPAR γ (** **≤** **0.300)**	0.965	0.297	0.022	0.763	0.021	0.017	0.964	0.196	0.020	0.826	0.019	0.021
**SR-ARE (** **≤** **0.300)**	0.556	0.275	0.064	0.200	0.081	0.049	0.637	0.216	0.049	0.163	0.058	0.038
**SR-ATAD5 (** **≤** **0.300)**	0.052	0.118	0.008	0.078	0.040	0.009	0.428	0.385	0.015	0.190	0.148	0.016
**SR-HSE (** **≤** **0.300)**	0.747	0.128	0.027	0.567	0.050	0.030	0.770	0.211	0.031	0.507	0.091	0.041
**SR-MMP (** **≤** **0.300)**	0.971	0.942	0.647	0.940	0.876	0.620	0.956	0.808	0.261	0.806	0.469	0.277
**SR-p53 (** **≤** **0.300)**	0.271	0.130	0.010	0.443	0.112	0.012	0.647	0.432	0.011	0.537	0.353	0.015
**Acute Toxicity Rule A.**	0	0	0	0	0	0	0	0	0	0	0	0
**Gen. Carcin. Rule A.**	0	0	0	0	0	0	0	0	0	0	0	0
**Non Gen. Carcin. Rule A.**	0	0	0	0	0	0	0	0	0	0	0	0
**Skin Sensit. Rule A.**	0	0	0	0	0	0	0	0	0	0	0	0
**Aquatic Tox. Rule A.**	1	1	2	1	1	1	0	0	1	0	0	0
**Non Biodegr. Rule A.**	0	0	1	0	0	0	0	0	1	0	0	0
**SureChEMBL Rule**	0	0	0	0	0	0	0	0	0	0	0	0
**FAF-Drugs4 Rule**	0	0	0	0	0	0	0	0	0	0	0	0
**Toxicophores**	0	0	0	0	0	0	0	0	0	0	0	0

**Legend**: **A.** = alerts; **Bioconc.** = bioconcentration; **Biodegr.** = biodegradation; **carcin.** = carcinogenicity; **Gen. Carcin.** = genotoxic carcinogenicity; **inh.** = inhibitor; **Mol.** = molecular; **penetr.** = penetration; **perm.** = permeability; **Respir. Tox.** = respiratory toxicity; **ROA** = rat oral acute toxicity; **sensit.** = sensitization; **sub.** = substrate; **Tox.** = toxicity; **for values given in a range 0.000**–**1.000** (unless stated otherwise): **0.000**–**0.300** = low probability, **0.301–0.699** = moderate probability, **0.700**–**1.000** = high probability.

**Table 10:** Acetylation of triterpenes (on the example of oleanolic acid) as a method of improving cytotoxic activity.

Results		Ref.
Oleanolic acid: IC_50_ = 6.4 µM (A-549)	Acetyloleanolic acid: IC_50_ = 5.8 µM (A-549)	[104]
Oleanolic acid: IC_50_ > 10 µM (KB)	Acetyloleanolic acid: IC_50_ = 7.6 µM (KB)	
Oleanolic acid: IC_50_ > 10 µM (KB-VIN)	Acetyloleanolic acid: IC_50_ = 7.6 µM (KB-VIN)	
Oleanolic acid: IC_50_ = 106.4 µM (B16-F10)	Acetyloleanolic acid: IC_50_ = 64.7 µM (B16-F10)	[105]
Oleanolic acid: IC_50_ = 429.9 µM (HT-29)	Acetyloleanolic acid: IC_50_ = 148.5 µM (HT-29)	
Oleanolic acid: IC_50_ = 211.8 µM (Hep-G2)	Acetyloleanolic acid: IC_50_ = 103.75 µM (Hep-G2)	
Oleanolic acid: IC_50_ = 41.7 µM (RAW 264.7)	Acetyloleanolic acid: IC_50_ = 13.8 µM (RAW 264.7)	[106]
Oleanolic acid: IC_50_ = 35.2 µM (J774A.1)	Acetyloleanolic acid: IC_50_ = 16.8 µM (J774A.1)	
Oleanolic acid: IC_50_ = 106.4 µM (B16-F10)	Acetyloleanolic acid: IC_50_ = 64.7 µM (B16-F10)	[107]

**Legend**: **IC_50_** = half maximal inhibitory concentration; **A-459** = *human lung carcinoma*; **KB** = *human oral squamous carcinoma*; **KB-VIN** = *multidrug resistant human epidermoid carcinoma*; **B16-F10** = *murine melanoma*; **HT-29** = *human colorectal adenocarcinoma*; **Hep-G2** = *human hepatocellular carcinoma*; **RAW 264.1** = adherent cell line isolated from a mouse tumor that was induced by Abelson murine leukemia virus; **J774A.1** = macrophage-like cell line derived from a BALB/c mouse.

## Data Availability

All data concerning this paper are available in the manuscript body or in the Supplementary Data.

## Supplementary Materials

The following supporting information can be downloaded at https://www.mdpi.com/article/10.3390/molecules30122661/s1: Supplementary Materials, Table S1.1. ^1^H NMR data for mother triterpenes **1a**–**6a** and their derivatives **1b**–**6b** were obtained via acetic anhydride and aspirin acylation. Table S1.2. ^13^C NMR data for mother triterpenes **1a**–**6a** and their derivatives **1b**–**6b** were obtained via acetic anhydride and aspirin acylation. Table S2. Docking result for compounds **1a**–**6a** and **1b**–**6b** with p53 protein Y220.
