# Supplementary material for: Enhancing the Pharmacological Properties of Triterpenes Through Acetylation: An Anticancer and Antioxidant Perspective
Source: Molecules. 2025 Jun 19;30(12):2661. doi: 10.3390/molecules30122661 (PMC12196235; doi:10.3390/molecules30122661)
Supplement: Supplementary file 1 [file molecules-30-02661-s001.zip › molecules-3570419-supplementary.pdf]

Article

Enhancing the Pharmacological Properties of Triterpenes Through Acetylation: An Anticancer and Antioxidant Perspective

Barbara Bednarczyk-Cwynar [1,2,*], Piotr Ruszkowski [3], Andrzej Günther [1], Szymon Sip [4], Katarzyna Bednarek-Rajewska [5] and Przemysław Zalewski [4]

[1] Department of Organic Chemistry, Faculty of Pharmacy, Poznan University of Medical Sciences, Collegium Pharmaceuticum 2, (CP.2), Rokietnicka Str. 3, 60–806 Poznan, Poland; bcwynar@ump.edu.pl; andrzej.gunther@me.pl

[2] Center of Innovative Pharmaceutical Technology (CITF), Rokietnicka Str. 3, 60–806 Poznan, Poland; bcwynar@ump.edu.pl;

[3] Department of Pharmacology, Faculty of Pharmacy, Poznan University of Medical Sciences, Collegium Pharmaceuticum 1 (CP.1), Rokietnicka Str. 3, 60–806 Poznan, Poland; pruszkowski@gmail.com

[4] Department of Pharmacognosy and Biomaterials, Faculty of Pharmacy, Poznan University of Medical Sciences, Collegium Pharmaceuticum 1 (CP.1), Rokietnicka Str. 3, 60–806 Poznan, Poland; pzalewski@ump.edu.pl; szymonsip@ump.edu.pl

[5] Department of Clinical Pathology, Faculty of Medical Sciences, Poznan University of Medical Sciences, Przybyszewskiego Str. 49, 60-355 Poznan, Poland, krajewska@ump.edu.pl

* Correspondence: Barbara Bednarczyk-Cwynar, bcwynar@ump.edu.pl

Supplementary Materials. Table of contents

Table S1.1. ^{1}H NMR data for mother triterpenes **1a** – **6a** and their derivatives **1b** – **6b** obtained *via* acetic anhydride and *via* aspirin acylation.
Table S1.2. ^{13}C NMR data for mother triterpenes **1a** – **6a** and their derivatives **1b** – **6b** obtained *via* acetic anhydride and *via* aspirin acylation.
Table S2. Docking result for compounds **1a** – **6a** and **1b** – **6b** with p53 protein Y220.

Table S1.1. ^{1}H NMR data for mother triterpenes **1a – 6a** and their derivatives **1b – 6b** obtained *via* acetic anhydride and *via* aspirin acylation.

s or br/s	d	t	dd	dt	td	m

mother triterpene		3-*O*-acetyl derivative obtained *via* acetic anhydride acylation (Procedure D)		3-*O*-acetyl derivative obtained *via* aspirin acylation (Procedure I)		Assignment
**TT no.**	**chemical shift, δ [ppm], *J* [Hz]**	**TT no.**	**chemical shift, δ [ppm], *J* [Hz]**	**TT no.**	**chemical shift, δ [ppm], *J* [Hz]**	
**1a**	11.80 (1H, br/s)	**1b**	11.80 (1H, br/s)	**1b**	11.81 (1H, br/s)	COOH
	5.27 (1H, t, *J* = 3.4)		5.26 (1H, t, *J* = 3.1)		5.27 (1H, t, *J* = 3.3)	C_{12}-H
	3.22 (1H, dd, *J* = 4.8, 10.6)		4.49 (1H, t, *J* = 7.9)		4.50 (1H, t, *J* = 7.9)	C_3-H_α
	2.83 (1H, t, *J* = 7.1)		2.82 (1H, dd, *J* = 3.8, 13.2)		2.82 (1H, dd, *J* = 3.8, 13.5)	C_{18}-H_β
	-		2.05 (3H, s)		2.05 (3H, s)	CH_3COO
	1.30, 0.98, 0.92, 0.91, 0.90, 0.77, 0.74 (7 x 3H, 7 x s)		1.12, 0.94, 0.92, 0.90, 0.86, 0.85, 0.74 (7 x 3H, 7 x s)		1.12, 0.94, 0.93, 0.90, 0.86, 0.85, 0.74 (7 x 3H, 7 x s)	C_{23}-H_3 – C_{27}-H_3, C_{29}-H_3, C_{30}-H_3
**2a**	5.19 (1H, t, *J* = 3.4)	**2b**	5.20 (1H, t, *J* = 3.4)	**2b**	5.20 (1H, t, *J* = 3.6)	C_{12}-H
	3.70 (1H, m)		4.50 (1H, t, *J* = 8.0)		4.49 (1H, t, *J* = 8.0)	C_3-H_α
	3.55 (1H, d, *J* = 11.0)		4.03 (1H, d, *J* = 11.0)		4.03 (1H, d, *J* = 11.0)	C_{28}-H_a
	3.20 (1H, d, *J* = 10.7)		3.70 (1H, d, *J* = 11.0)		3.69 (1H, d, *J* = 11.2)	C_{28}-H_b
	-		2.05 (3H, s) and 2.04 (3H, s)		2.06 (3H, s) and 2.05 (3H, s)	CH_3COO and $OCOCH_3$
	1.16, 0.99, 0.94, 0.93, 0.89, 0.87, 0.79 (7 x 3H, 7 x s)		1.16, 0.95, 0.94, 0.89, 0.88, 0.87, 0.86 (7 x 3H, 7 x s)		1.16, 0.95, 0.94, 0.89, 0.88, 0.87, 0.86 (7 x 3H, 7 x s)	C_{23}-H_3 – C_{27}-H_3, C_{29}-H_3, C_{30}-H_3
**3a**	3.77 (1H, dd, *J* = 1.2, 7.7)	**3b**	3.77 (1H, dd, *J* = 1.0, 7.6)	**3b**	3.77 (1H, dd, *J* = 1.0, 7.7)	C_{28}-H_a
	3.44 (1H, d, *J* = 7.7)		3.44 (1H, d, *J* = 7.8)		3.44 (1H, d, *J* = 7.8)	C_{28}-H_b
	3.53 (1H, s)		3.52 (1H, s)		3.53 (1H, s)	C_{19}-H
	3.19 (1H, dd, *J* = 5.1, 11.2)		4.48 (1H, dd, *J* = 6.1, 10.3)		4.48 (1H, dd, *J* = 6.1, 10.3)	C_3-H_α

	-		2.04 (3H, s)		2.04 (3H, s)	CH_3COO
	1.72 (1H, dt, *J* = 3.6, 13.0)		1.72 (1H, dt, *J* = 3.6, 13.1)		1.72 (1H, dt, *J* = 3.5, 13.1)	C_{18}-H_{β}
	0.98, 0.97, 0.93, 0.91, 0.84, 0.79, 0.76 (7 x 3H, 7 x s)		0.97, 0.93, 0.91, 0.87, 0.85, 0.84, 0.80 (7 x 3H, 7 x s)		0.97, 0.93, 0.91, 0.87, 0.85, 0.84, 0.80 (7 x 3H, 7 x s)	C_{23}-H_3 – C_{27}-H_3, C_{29}-H_3, C_{30}-H_3
	-		11.57 (1H, br/s)		11.55 (1H, br/s)	COOH
	4.74 (1H, d, *J* = 2.0)		4.73 (1H, d, *J* = 1.4)		4.74 (1H, d, *J* = 1.4)	C_{29}-H_a
	4.62 (1H, d, *J* = 1.3)		4.60 (1H, s)		4.61 (1H, s)	C_{29}-H_b
	3.19 (1H, dd, *J* = 5.0, 11.3)		4.47 (1H, dd, *J* = 5.8, 10.3)		4.47 (1H, dd, *J* = 5.8, 10.3)	C_3-H_{α}
**4a**	3.00 (1H, td, *J* = 4.6, 10.7)	**4b**	3.00 (1H, td, *J* = 4.6, 10.7)	**4b**	3.00 (1H, td, *J* = 4.6, 10.7)	C_{19}-H_{β}
	2.28 (1H, d, *J* = 12.8)		2.29 (1H, d, *J* = 12.8)		2.28 (1H, d, *J* = 12.8)	C_{18}-H
	-		2.03 (3H, s)		2.04 (3H, s)	CH_3COO
	1.69 (3H, s)		1.69 (3H, s)		1.69 (3H, s)	C_{30}-H_3
	0.98, 0.96, 0.93, 0.82, 0.75 (5 x 3H, 5 x s)		0.98, 0.93, 0.85, 0.84, 0.83 (5 x 3H, 5 x s)		0.97, 0.93, 0.85, 0.84, 0.83 (5 x 3H, 5 x s)	C_{23}-H_3 – C_{27}-H_3
	4.68 (1H, d, *J* = 2.3)		4.69 (1H, d, *J* = 2.1)		4.69 (1H, d, *J* = 1.9)	C_{29}-H_a
	4.58 (1H, dd, *J* = 1.1, 2.2)		4.59 (1H, dd, *J* = 1.4, 2.2)		4.59 (1H, dd, *J* = 1.4, 2.1)	C_{29}-H_b
	3.18 (1H, dd, *J* = 5.1, 10.8)		4.46 (1H, dd, *J* = 5.6, 10.3)		4.47 (1H, dd, *J* = 5.7, 10.3)	C_3-H_{α}
	3.80 (1H, dd, *J* = 1.2, 10.7)		4.25 (1H, dd, *J* = 1.3, 11.1)		4.25 (1H, dd, *J* = 1.3, 11.0)	C_{28}-H_a
**5a**	3.33 (1H, d, *J* = 10.8)	**5b**	3.85 (1H, d, *J* = 11.0)	**5b**	3.85 (1H, d, *J* = 11.0)	C_{28}-H_b
	2.44 (1H, td, *J* = 5.8, 10.9)		2.44 (1H, td, *J* = 5.8, 10.9)		2.45 (1H, td, *J* = 5.8, 10.8)	C_{19}-H
	-		2.07 (3H, s) and 2.04 (3H, s)		2.07 (3H, s) and 2.04 (3H, s)	CH_3COO and $OCOCH_3$
	1.68 (3H, s)		1.68 (3H, s)		1.68 (3H, s)	C_{30}-H_3
	1.02, 0.98, 0.97, 0.82, 0.76 (5 x 3H, 5 x s)		1.03, 0.97, 0.84 x 2, 0.83 (5 x 3H, 5 x s)		1.03, 0.97, 0.84 x 2, 0.83 (5 x 3H, 5 x s)	C_{23}-H_3 – C_{27}-H_3
**6a**	4.69 (1H, d, *J* = 2.4)	**6b**	4.68 (1H, d, *J* = 2.4)	**6b**	4.68 (1H, d, *J* = 2.4)	C_{29}-H_a
	4.56 (1H, dd, *J* = 1.5, 2.5)		4.57 (1H, dd, *J* = 1.4, 2.4)		4.57 (1H, dd, *J* = 1.5, 2.4)	C_{29}-H_b

	3.18 (1H, dd, *J* = 5.0, 11.3)		4.47 (1H, dd, *J* = 6.1, 10.1)		4.47 (1H, dd, *J* = 5.8, 10.4)	C_3-H_α
	2.38 (1H, dt, *J* = 5.8, 11.0)		2.37 (1H, dt, *J* = 5.8, 11.0)		2.37 (1H, dt, *J* = 5.8, 11.0)	C_{19}-H
	-		2.04 (3H, s)		2.04 (3H, s)	CH_3COO
	1.97 – 1.86 (1H, m)		1.97 – 1.86 (1H, m)		1.99 – 1.86 (1H, m)	C_{18}-H_β
	1.68 (3H, s)		1.68 (3H, s)		1.68 (3H, s)	C_{30}-H
	1.03, 0.96, 0.94, 0.83, 0.79 (5 x 3H, 5 x s)		1.03, 0.94, 0.85, 0.84, 0.83 (5 x 3H, 5 x s)		1.03, 0.94, 0.85, 0.84, 0.83 (5 x 3H, 5 x s)	C_{23}-H_3 – C_{27}-H_3
	0.77 (3H, s)		0.78 (3H, s)		0.78 (3H, s)	C_{28}-H_3

Table S1.2. ^{13}C NMR data for mother triterpenes **1a – 6a** and their derivatives **1b – 6b** obtained *via* acetic anhydride and *via* aspirin acylation.

CH_3	CH_2	CH	C_q

mother triterpene		3-*O*-acetyl derivative obtained *via* acetic anhydride acylation (Procedure D)		3-*O*-acetyl derivative obtained *via* aspirin acylation (Procedure I)		assignment
**TT no.**	**chemical shift, δ [ppm]**	**TT no.**	**chemical shift, δ [ppm]**	**TT no.**	**chemical shift, δ [ppm]**	
**1a**	183.6 (C_q)	**1b**	183.9 (C_q)	**1b**	184.5 (C_q)	C-28
	-		170.7 (C_q)		171.0 (C_q)	CH_3COO
	143.6 (C_q)		143.5 (C_q)		143.6 (C_q)	C-13
	122.6 (CH)		122.5 (CH)		122.5 (CH)	C-12
	79.0 (CH)		80.9 (CH)		80.9 (CH)	C-3
	46.5 (C_q)		46.6 (C_q)		46.5 (C_q)	C-17
	41.0 (CH)		41.0 (CH)		40.8 (CH)	C-18
	-		21.3 (CH_3)		21.3 (CH_3)	CH_3COO
**2a**	-	**2b**	171.3 (C_q) and 171.0 (C_q)	**2b**	171.3 (C_q) and 171.0 (C_q)	$OCOCH_3$ and CH_3COO

	144.2 (C_q)		143.6 (C_q)		143.5 (C_q)	C-13
	122.3 (CH)		122.8 (CH)		122.7 (CH)	C-12
	79.0 (CH)		80.9 (CH)		80.8 (CH)	C-3
	69.6 (CH_2)		70.7 (CH_2)		70.7 (CH_2)	C-28
	42.3 (CH)		42.4 (CH)		42.4 (CH)	C-18
	38.7 (C_q)		35.8 (C_q)		35.7 (C_q)	C-17
	-		21.2 (CH_3) and 20.9 (CH_3)		21.3 (CH_3) and 20.9 (CH_3)	CH_3COO and $OCOCH_3$
	-		170.9 (C_q)		170.9 (C_q)	CH_3COO
	87.9 (CH)		87.9 (CH)		87.9 (CH)	C-19
	78.8 (CH)		80.8 (CH)		80.8 (CH)	C-3
**3a**	71.2 (CH_2)	**3b**	71.2 (CH_2)	**3b**	71.2 (CH_2)	C-28
	46.7 (CH)		46.8 (CH)		46.7 (CH)	C-18
	36.2 (C_q)		36.2 (C_q)		36.2 (C_q)	C-17
	-		21.3 (CH_3)		21.3 (CH_3)	CH_3COO
	182.8 (C_q)		182.8 (C_q)		182.8 (C_q)	C-28
	-		171.1 (C_q)		171.1 (C_q)	CH_3COO
	150.4 (C_q)		150.4 (C_q)		150.3 (C_q)	C-20
	109.7 (CH_2)		109.7 (CH_2)		109.7 (CH_2)	C-29
**4a**	79.0 (CH)	**4b**	80.9 (CH)	**4b**	80.9 (CH)	C-3
	50.5 (CH)		50.4 (CH)		50.3 (CH)	C-18
	49.2 (CH)		49.2 (CH)		49.2 (CH)	C-19
	42.4 (C_q)		42.3 (C_q)		42.3 (C_q)	C-17
	-		21.3 (CH_3)		21.3 (CH_3)	CH_3COO
	19.3 (CH_3)		19.3 (CH_3)		19.3 (CH_3)	C-30

	-		171.5 (C_q) and 170.9 (C_q)		171.6 (C_q) and 171.0 (C_q)	$OCOCH_3$ and CH_3COO
	150.5 (C_q)		150.1 (C_q)		150.1 (C_q)	C-20
	109.6 (CH_2)		109.8 (CH_2)		109.8 (CH_2)	C-29
	79.0 (CH)		80.9 (CH)		80.9 (CH)	C-3
**5a**	60.5 (CH_2)	**5b**	62.7 (CH_2)	**5b**	62.7 (CH_2)	C-28
	48.7 (CH)		48.7 (CH)		48.7 (CH)	C-19
	47.8 (CH)		47.6 (CH)		47.7 (CH)	C-18
	47.7 (C_q)		46.2 (C_q)		46.3 (C_q)	C-17
	-		21.0 (CH_3) and 20.9 (CH_3)		21.3 (CH_3) and 21.0 (CH_3)	$OCOCH_3$ and CH_3COO
	19.1 (CH_3)		19.1 (CH_3)		19.1 (CH_3)	C-30
	-		170.9 (C_q)		170.9 (C_q)	CH_3COO
	150.9 (C_q)		150.9 (C_q)		150.8 (C_q)	C-20
	109.3 (CH_2)		109.3 (CH_2)		109.3 (CH_2)	C-29
	78.9 (CH)		80.9 (CH)		80.9 (CH)	C-3
**6a**	48.2 (CH)	**6b**	48.2 (CH)	**6b**	48.2 (CH)	C-19
	47.9 (CH)		48.0 (CH)		47.9 (CH)	C-18
	42.9 (C_q)		42.8 (C_q)		42.9 (C_q)	C-17
	19.3 (CH_3)		19.3 (CH_3)		19.2 (CH_3)	C-30
	18.0 (CH_3)		18.0 (CH_3)		17.9 (CH_3)	C-28

Table S2. Docking result for compounds **1a**–**6a** and **1b**–**6b** with p53 protein Y220.

Compound No	CurPocket ID	Vina score	Cavity volume (Å^3)	Center (x, y, z)	Docking size (x, y, z)
	C1	-6.4	3942	-16, 49, 61	35, 23, 32
	C2	-6.3	2320	-6, 46, 9	23, 23, 23
**1a**	C3	-8.6	828	-33, 65, 34	23, 23, 23
	C4	-8.2	430	-40, 55, 40	23, 23, 23
	C5	-7.8	370	-30, 44, 24	23, 23, 23
	C1	-9.0	3942	-16, 49, 61	35, 23, 32
	C2	-9.0	2320	-6, 46, 9	23, 23, 23
**1b**	C3	-7.4	828	-33, 65, 34	23, 23, 23
	C4	-8.5	430	-40, 55, 40	23, 23, 23
	C5	-7.8	370	-30, 44, 24	23, 23, 23
	C1	-7.6	3942	-16, 49, 61	35, 23, 32
	C2	-6.5	2320	-6, 46, 9	23, 23, 23
**2a**	C3	-9.3	828	-33, 65, 34	23, 23, 23
	C4	-8.0	430	-40, 55, 40	23, 23, 23
	C5	-7.5	370	-30, 44, 24	23, 23, 23
	C1	-7.1	3942	-16, 49, 61	35, 25, 32
	C2	-6.4	2320	-6, 46, 9	25, 25, 25
**2b**	C3	-10.1	828	-33, 65, 34	25, 25, 25
	C4	-8.8	430	-40, 55, 40	25, 25, 25
	C5	-8.9	370	-30, 44, 24	25, 25, 25
	C1	-8.9	3942	-16, 49, 61	35, 22, 32
	C2	-6.5	2320	-6, 46, 9	22, 28, 22
**3a**	C3	-9.9	828	-33, 65, 34	22, 22, 22
	C4	-9.4	430	-40, 55, 40	22, 22, 22
	C5	-8.3	370	-30, 44, 24	22, 22, 22
	C1	-8.3	3942	-16, 49, 61	35, 24, 32
	C2	-6.4	2320	-6, 46, 9	24, 24, 24
**3b**	C3	-9.3	828	-33, 65, 34	24, 24, 24
	C4	-9.1	430	-40, 55, 40	24, 24, 24

	C5	-7.9	370	-30, 44, 24	24, 24, 24
	C1	-6.0	3942	-16, 49, 61	35, 22, 32
	C2	-6.2	2320	-6, 46, 9	22, 28, 22
**4a**	C3	-8.6	828	-33, 65, 34	22, 22, 22
	C4	-8.6	430	-40, 55, 40	22, 22, 22
	C5	-6.8	370	-30, 44, 24	22, 22, 22
	C1	-7.8	3942	-16, 49, 61	35, 23, 32
	C2	-5.7	2320	-6, 46, 9	23, 23, 23
**4b**	C3	-9.3	828	-33, 65, 34	23, 23, 23
	C4	-8.4	430	-40, 55, 40	23, 23, 23
	C5	-9.0	370	-30, 44, 24	23, 23, 23
	C1	-7.1	3942	-16, 49, 61	35, 23, 32
	C2	-5.8	2320	-6, 46, 9	23, 23, 23
**5a**	C3	-8.2	828	-33, 65, 34	23, 23, 23
	C4	-8.0	430	-40, 55, 40	23, 23, 23
	C5	-7.1	370	-30, 44, 24	23, 23, 23
	C1	-9.1	3942	-16, 49, 61	35, 23, 32
	C2	-6.0	2320	-6, 46, 9	23, 23, 23
**5b**	C3	-8.1	828	-33, 65, 34	23, 23, 23
	C4	-8.2	430	-40, 55, 40	23, 23, 23
	C5	-7.5	370	-30, 44, 24	23, 23, 23
	C1	-8.5	3942	-16, 49, 61	35, 22, 32
	C2	-6.0	2320	-6, 46, 9	22, 28, 22
**6a**	C3	-9.3	828	-33, 65, 34	22, 22, 22
	C4	-9.6	430	-40, 55, 40	22, 22, 22
	C5	-7.5	370	-30, 44, 24	22, 22, 22
	C1	-9.1	3942	-16, 49, 61	35, 23, 32
	C2	-6.1	2320	-6, 46, 9	23, 23, 23
**6b**	C3	-9.6	828	-33, 65, 34	23, 23, 23
	C4	-8.6	430	-40, 55, 40	23, 23, 23
	C5	-7.2	370	-30, 44, 24	23, 23, 23
